# Extracellular aggregated alpha synuclein primarily triggers lysosomal dysfunction in neural cells prevented by trehalose

**DOI:** 10.1038/s41598-018-35811-8

**Published:** 2019-01-24

**Authors:** Anna-Carin Hoffmann, Georgia Minakaki, Stefanie Menges, Rachele Salvi, Sergey Savitskiy, Aida Kazman, Hugo Vicente Miranda, Dirk Mielenz, Jochen Klucken, Jürgen Winkler, Wei Xiang

**Affiliations:** 1 Institute of Biochemistry, Friedrich-Alexander-University Erlangen-Nürnberg (FAU), Erlangen, Germany; 2 Division of Molecular Neurology, University Hospital Erlangen, FAU Erlangen-Nürnberg, Erlangen, Germany; 3 Center for Human Genetics Regensburg, Regensburg, Germany; 4 Department of Chemistry, Technical University of Munich, Munich, Germany; 5 CEDOC, Chronic Diseases Research Center, NOVA Medical School | Faculdade de Ciências Médicas, Universidade NOVA de Lisboa, Campo dos Mártires da Pátria, 130, 1169-056 Lisboa, Portugal; 6 Division of Molecular Immunology, Nikolaus-Fiebiger-Center, Department of Internal Medicine III, FAU Erlangen-Nürnberg, Erlangen, Germany

## Abstract

Cell-to-cell propagation of aggregated alpha synuclein (aSyn) has been suggested to play an important role in the progression of alpha synucleinopathies. A critical step for the propagation process is the accumulation of extracellular aSyn within recipient cells. Here, we investigated the trafficking of distinct exogenous aSyn forms and addressed the mechanisms influencing their accumulation in recipient cells. The aggregated aSyn species (oligomers and fibrils) exhibited more pronounced accumulation within recipient cells than aSyn monomers. In particular, internalized extracellular aSyn in the aggregated forms was able to seed the aggregation of endogenous aSyn. Following uptake, aSyn was detected along endosome-to-lysosome and autophagosome-to-lysosome routes. Intriguingly, aggregated aSyn resulted in lysosomal activity impairment, accompanied by the accumulation of dilated lysosomes. Moreover, analysis of autophagy-related protein markers suggested decreased autophagosome clearance. In contrast, the endocytic pathway, proteasome activity, and mitochondrial homeostasis were not substantially affected in recipient cells. Our data suggests that extracellularly added aggregated aSyn primarily impairs lysosomal activity, consequently leading to aSyn accumulation within recipient cells. Importantly, the autophagy inducer trehalose prevented lysosomal alterations and attenuated aSyn accumulation within aSyn-exposed cells. Our study underscores the importance of lysosomes for the propagation of aSyn pathology, thereby proposing these organelles as interventional targets.

## Introduction

Alpha synucleinopathies, including Parkinson’s disease (PD), dementia with Lewy bodies, and multiple system atrophy, are characterized by intracellular deposition of alpha synuclein (aSyn)^1–3^. It is widely accepted that abnormal aggregation of aSyn, a physiologically soluble protein with a molecular weight of 14 kDa, contributes to the neurodegeneration in alpha synucleinopahties. Current knowledge about aSyn aggregation suggests that aSyn monomers are first assembled into oligomers and subsequently into β-sheet-rich amyloid fibrils^2,4^. Amyloid fibrils are finally deposited along with other components, forming inclusions, such as the Lewy bodies.

In addition to pathological aSyn aggregation, mitochondrial dysfunction and impaired protein degradation pathways, including the autophagy-lysosomal pathway (ALP) and the ubiquitin-proteasome system, have been linked to the neurodegeneration in alpha synucleinopathies^5–7^. Moreover, cell-to-cell propagation of pathogenic aSyn was recently suggested to be a mechanism contributing to the progression of alpha synucleinopathies. The propagation hypothesis was initially based on the clinical and neuropathological findings that (1) aSyn was detected in blood plasma and cerebrospinal fluid^8,9^; (2) the distribution of aggregated aSyn in postmortem brains of PD patients correlated with the clinical stages of patients^10^, suggesting a progressive spreading of aSyn pathology between brain regions; (3) embryonic mesencephalic neurons grafted into the neostriatum of PD patients developed Lewy bodies^11,12^.

A cell-to-cell propagation pathway implies that aggregated aSyn is released from cells, uptaken by neighboring cells, and stimulates the aggregation of endogenous aSyn within recipient cells, probably serving as a “seed” of further aggregation processes. Consequently, the spreading of aggregated aSyn between cells not only induces the propagation of neurotoxic aSyn species, but also triggers the pathology in recipient cells. While numerous studies have been carried out in the past few years to recapitulate and to verify the propagation of aSyn pathology, e.g. by using aSyn preformed fibrils^13,14^, the precise mechanistic pathways of aSyn propagation between cells remain vague.

For achieving cell-to-cell propagation, it is crucial that internalized extracellular aSyn bypasses the protein degradation pathways, such as ALP and ubiquitin-proteasome system, accumulates within recipient cells, and finally interacts with endogenous aSyn and other cellular targets. Understanding the trafficking and accumulation of extracellular aSyn within recipient cells is not only important for clarifying the role of aSyn propagation in neurodegeneration, but also for identifying novel targets for intervention. Here, we investigated the trafficking behavior of extracellularly added aSyn in different aggregation states and characterized the target pathways in recipient cells. We observed that extracellularly added aggregated aSyn was processed in recipient cells considerably different from monomeric aSyn. In addition, we identified lysosomes and the ALP to be primarily affected upon exposure to aggregated aSyn. We further found that activation of lysosomal function by trehalose significantly prevents aSyn pathology in recipient cells.

## Results

### Aggregated aSyn species exhibit a stronger accumulation in recipient cells and are more efficiently uptaken than monomers

**Figure 1:**
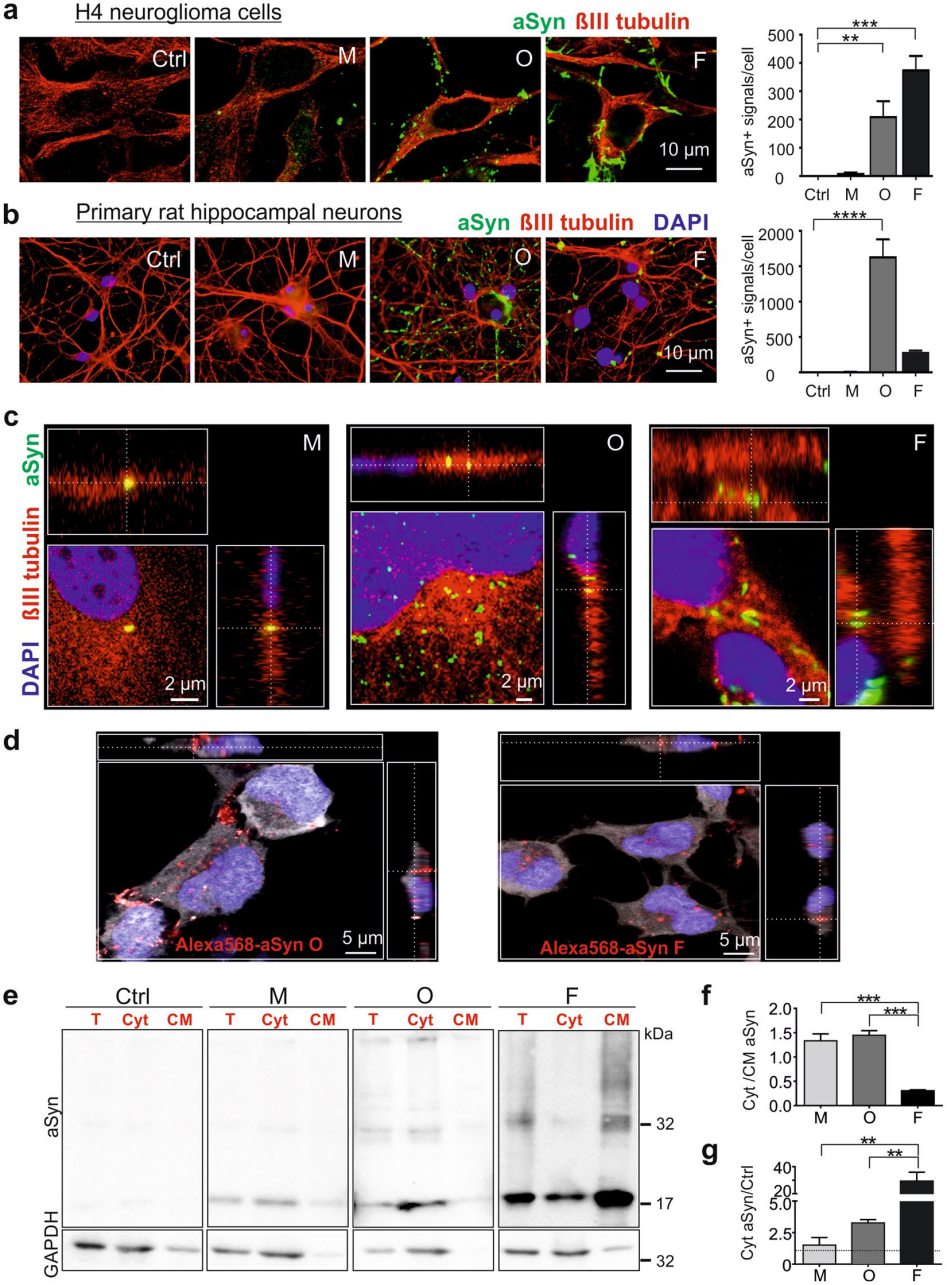
Accumulation of extracellular aSyn species in recipient cells and uptake of extracellular aSyn. Cells were incubated either without aSyn (Ctrl) or with monomeric (M), oligomeric (O), and fibrillar (F) aSyn. (**a**,**b)** Representative fluorescent confocal images of H4 cells (**a**) and primary rat hippocampal neurons (**b**) labeled with βIII tubulin (red) and aSyn (green). Quantification of ICC (right) shows a stronger co-labeling of extracellularly added aggregated aSyn species with recipient cells. For quantification, the counts of aSyn immunopositive signal/cell were determined (n = 3, One-way ANOVA with Dunnett’s multiple comparisons test). (**c)** ICC and Z-stack images of aSyn-exposed H4 cells. Immunosignals of exogenous aSyn (green) were partly inside cells visualized by βIII tubulin (red). **(d)** ICC and Z-stack images of H4 cells exposed to AlexaFluor568-labeled aSyn oligomers (left) and fibrils (right). Labeled aSyn, βIII tubulin, and DAPI are present in red, white, and blue, respectively. (**e)** Representative WB images of exogenous aSyn in total cell homogenate (T), in the cytosol (Cyt), and in cellular membrane (CM) fraction of exposed H4 cells show the presence of exogenous aSyn in the cytosol. Lanes of aSyn and GAPDH within one frame (T, Cyt, and CM) are derived from the same blot. aSyn and GAPDH in T, Cyt, and CM fractions of each sample were probed in the identical blot. In each experiment, samples on different gels and blots were processed in parallel under same SDS-PAGE and immunodetection conditions, and exposed together for chemiluminescent detection. (**f)** Analysis of the proportion of aSyn distributed in cytosolic (Cyt) and associated with cellular membrane (CM) by calculating the ratio of aSyn levels in Cyt and CM fractions. (**g**) Relative aSyn levels in the cytosol of aSyn-exposed cells  normalized to those of control cells  (n = 3, One-way ANOVA with Tukey’s multiple comparisons test).

To address whether the uptake efficiency of aSyn differs between its aggregation states, we first analyzed the accumulation of extracellularly added aSyn in human neuroglioma (H4) cells exposed to unlabeled aSyn monomers as well as preformed oligomers and fibrils. Due to the possibility that aSyn species may change their assembly after adding to cells, we use the term “extracellular aSyn”, indicating aSyn in the extracellular spaces and extracellularly added aSyn species in their original aggregation states, and the term “exogenous aSyn”, referring to aSyn that accumulates or is internalized in recipient cells. H4 cells have very low endogenous aSyn expression levels (Fig. 1a, Ctrl), allowing to follow intracellular trafficking routes of exogenous aSyn. In order to exclude massive cell death potentially related to extracellularly added aSyn, we assessed the viability of H4 cells exposed to aSyn of 1, 5, and 10 µM for 24 h. Cell viability was not significantly compromised by as high as 10 µM aSyn (Supplementary Fig. S1). As aSyn doses between 0.5–5 µM have been widely used in various cell models for aSyn transfer and the concentration of aSyn at the synapse is expected to be in the range of 2–5 µM^15–18^, we used 1 µM aSyn for 24 h in the treatments throughout the study, unless otherwise stated.

Immunocytochemical (ICC) analysis demonstrated that all extracellularly added aSyn species accumulate in H4 recipient cells, albeit to a considerably different extent. Specifically, the accumulation of extracellular aSyn in H4 cells was enhanced with increasing aggregation states, in the order of fibrils > oligomers > monomers (Fig. 1a). As the expression of aSyn gene was unaltered in aSyn-exposed cells (Supplementary Fig. S2, left), the accumulation of aSyn immunosignal was mainly attributed to exogenous aSyn. The same analysis for H4 cells was also performed for primary rat hippocampal neurons using a human specific aSyn antibody (15G7). In primary neurons, we confirmed a stronger accumulation of the aggregated aSyn species (oligomers and fibrils) as compared to monomeric aSyn in recipient cells (Fig. 1b).

Since the loss of dopaminergic neurons and aSyn-containing inclusions in oligodendrocytes are hallmarks of PD and multiple system atrophy, respectively, we additionally tested the accumulation of exogenous aSyn in differentiated Lund human mesencephalic cells (LUHMES), which exhibit the phenotypes of dopaminergic neurons^19^, and in rat oligodendrocytic CG4 cells (Supplementary Fig. S3). Consistently, we detected a more pronounced increase of aSyn immunosignals in oligomer- or fibril-exposed cells than in monomer-exposed cells, indicating a stronger accumulation of the aggregated aSyn species in recipient cells. Overall, this aggregation-dependent accumulation pattern appeared to be common in different cell types.

We next determined the uptake of extracellularly added aSyn by confocal microscopic analysis of aSyn-exposed H4 cells. Z-stack confocal micrographs revealed that extracellular aSyn enters the exposed cells (Fig. 1c). While monomer-exposed cells mostly presented exogenous aSyn in the cytoplasm, oligomer- or fibril-exposed cells, were partially in the cytoplasm. To prove the uptake of aggregated species, we also applied AlexaFluor568-labeled oligomers and fibrils to H4 and confirmed the intracellular localization of the  exogenous aggregated aSyn species (Fig. 1d). Furthermore, we examined aSyn from both cytosolic and membrane fractions after a biochemical fractionation of exposed H4 cells (Supplementary Fig. S4a,b) and Western blot (WB) immunodetection of aSyn. In cytosolic fractions, we detected aSyn of all species analyzed, however, to different extents (Fig. 1e). While the majority of exogenous aSyn was present in the cytosol of monomer- and oligomer-exposed cells, a considerable proportion of exogenous fibrillar aSyn was attached to the plasma membrane or intracellular vesicular membranes (Fig. 1e,f). Nevertheless, the overall amount of exogenous aSyn in the cytosol was significantly higher, when aggregated aSyn, particularly the fibrillar species, were applied (Fig. 1e,g).

Taken together, uptake and accumulation within recipient cells was more prominent for the aggregated aSyn species as compared to monomeric aSyn.

### Administration of extracellular aggregated aSyn induces the aggregation of intracellular aSyn

**Figure 2:**
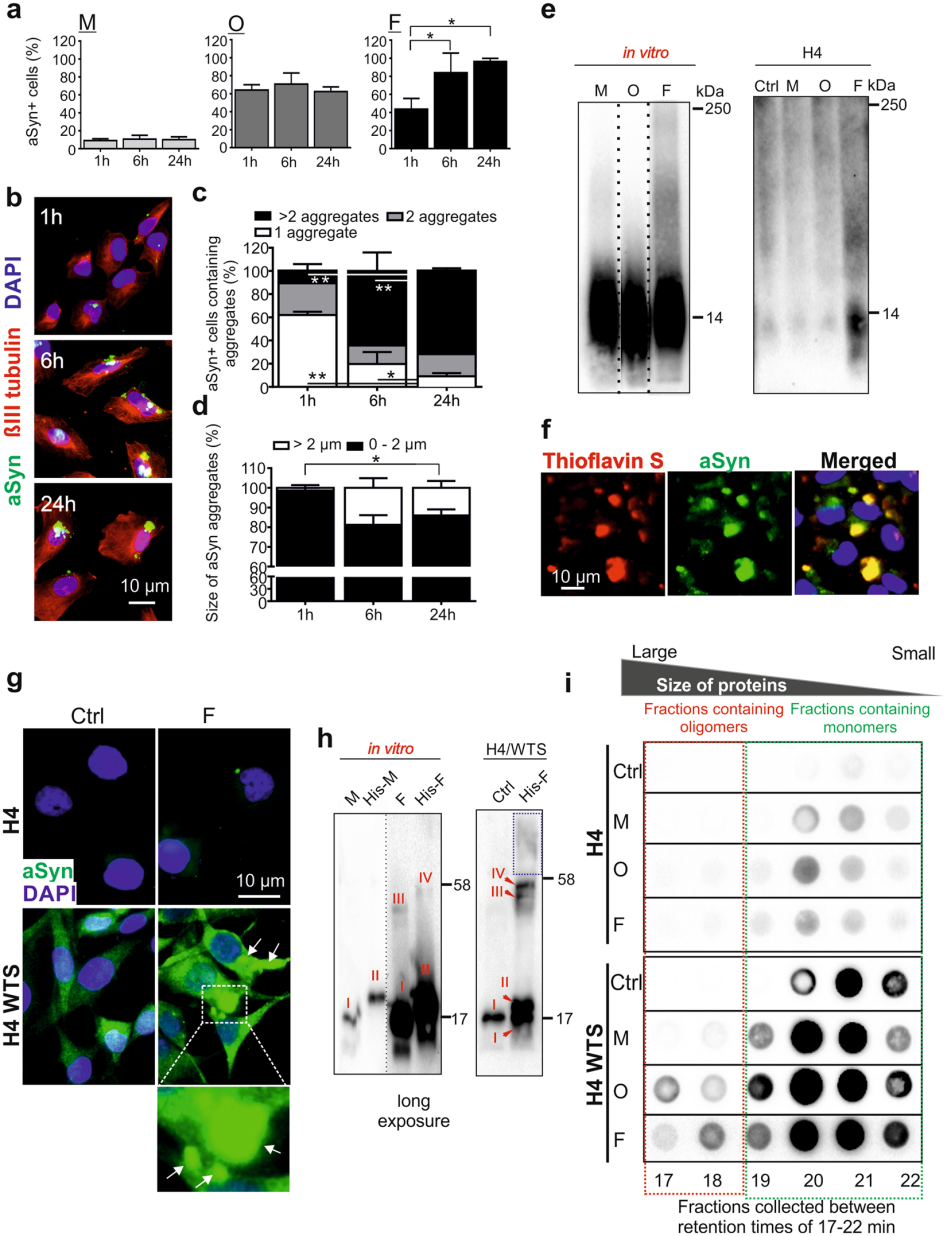
Stability and seeding effect of extracellular aggregated aSyn. (**a**) H4 cells were exposed to monomers (M, 2.5 µM), oligomers (O, 2.5 µM), and fibrils (F, 0.1 µM) for 1, 6, and 24 h, respectively. The proportion of aSyn+ cells were analyzed by ICC (n = 3, One-way ANOVA with Tukey’s multiple comparisons test). (**b)** Example images of fibril-exposed cells. (**c**,**d)** Percentage of aSyn+ cells carrying different numbers (1, 2, or >2, (**c**)) and different sizes of aSyn aggregates (diameter 0–2 µm or <2 µm, (**d**)) (n = 3, Two-way ANOVA with Tukey’s multiple comparisons test). **(e)** Blue native PAGE of aSyn species (*in vitro*, left**)** and cell lysates from H4 cells exposed to aSyn species (right). Blots within one black frame are derived from the same membrane. Lanes from different parts of the same membrane are separated by dashed lines. As the Syn1-antibody produced a stronger immunosignal for native monomers *in vitro*, different exposure times were used for aSyn species *in vitro* (left panel), allowing a clear comparison of the proportion of high molecular weight complexes in different species. (**f)** Overlap of fluorescent signals of extracellular aSyn (green) and Thioflavin S (red) in fibril-exposed H4 cells. (**g)** ICC of untreated H4 cells and H4/WTS cells (Ctrl), and cells treated with 0.1 µM fibrils for 1 h. Fibril-induced aSyn aggregation in H4/WTS cells is highlighted either by an enlarged view or by arrows in the ICC images. (**h)** SDS-PAGE of unlabeled aSyn monomers (M), fibrils (F), as well as histidine-tagged monomers (His-M) and fibrils (His-F). The aSyn species *in vitro* (left) and in H4/WTS (Ctrl or exposed to His-F for 6 h, right) can be recognized according to molecular weights (I: M, II: His-M, III: an oligomeric species of unlabeled aSyn, and IV: the corresponding oligomeric species of His-aSyn). To visualize species III and IV, a long exposure time was used. Blots within one black frame are derived from the same membrane. The lanes detected with different exposure times are separated by a dashed line. (**i)** SEC and dot blot analysis of cytosolic soluble proteins from H4 and H4/WTS cells exposed to aSyn species for 6 h. Signals within one black box (retention times: 17–22 min) were derived from the same dot blot membrane. Red and green frames highlight oligomer- and monomer- enriched fractions, respectively.

We next addressed the stability of exogenous aSyn in H4 recipient cells over time and examined its temporal accumulation pattern after exposure times of 1, 6, and 24 h via ICC analysis. As shown in Fig. 1a, fibrillar aSyn induced especially stronger aSyn immunosignals when using 1 µM for 24 h, while the signal intensity of monomeric aSyn was very low. In order to measure potential temporal changes of each specific species between 1–24 h within recipient cells via ICC, we used different aSyn concentrations for this set of exposure experiments (monomers and oligomers: 2.5 µM; fibrils: 0.1 µM). ICC analysis revealed that the proportion of aSyn immunopositive cells (aSyn+) was higher, when aggregated aSyn was applied (oligomers: 62–70% aSyn+ cells, fibrils: 43–96% aSyn+ cells) than monomeric aSyn (9–10% aSyn+ cells, even at a concentration of 2.5 µM) (Fig. 2a). Particularly, fibrils induced a time-dependent, proportional increase of aSyn+ cells. The number of aggregates per aSyn+ cell (Fig. 2b,c) and the size of aggregates were also enhanced over time (Fig. 2b,d).

To answer the question to which extent the aggregated aSyn species were conserved upon uptake, we first analyzed the patterns of aSyn species on blue native PAGE (Fig. 2e). We observed strong immunosignals above 14 kDa in fibril-exposed cells (right panel). Preformed fibrils *in vitro* also showed a high molecular weight smear (>14 kDa, left panel). In agreement, fluorescence microscopy of fibril-treated H4 cells demonstrated a signal overlap between aSyn and Thioflavin S, a dye that binds to amyloid fibrils (Fig. 2f). In contrast to fibrils, oligomers did not exhibit a notably different pattern in comparison to monomers on blue native PAGE, neither *in vitro* (Fig. 2e, left) nor in cells (Fig. 2e right). It is possible that the oligomeric species could not be clearly distinguished from the monomers on the native gel, when comparing their *in vitro* patterns (Fig. 2e, left). Alternatively, aSyn oligomers may disassemble during blue native PAGE. To address these issues, size exclusion chromatography (SEC) was complementarily performed, which has a higher resolution for monomers and oligomers (Supplementary Fig. S5a). We found that aSyn oligomers were mostly dissociated upon uptake by H4 cells (Supplementary Fig. S5b). Nevertheless, the level of oligomers (time of retention tR 12–17 min), although smaller than their original sizes (Supplementary Fig. 5a, *in vitro*, tR 10.4 min), was higher in oligomer-exposed cells than in monomer-exposed cells. Collectively, the results indicated that fibrillar aSyn conserves its structure, even after 24 h incubation, while oligomers are less stable and are degraded to a considerable extent to monomers or smaller oligomers.

Pronounced aSyn accumulation in oligomer- and fibril-exposed H4 cells raises the question of whether these aggregated aSyn species are able to increase the aggregation of endogenous aSyn. As naïve H4 cells express only a very low level of endogenous aSyn, we also used H4 cells stably overexpressing wild type aSyn (H4/WTS) for this experiment. We exposed the cells to fibrillar aSyn (0.1 µM) for 1 h, and analyzed the formation of aggregate-like structures by ICC as a readout of intracellular aSyn aggregation (Fig. 2g). In untreated H4/WTS cells, overexpressed endogenous aSyn is characterized by a diffuse staining (Fig. 2g, bottom left). In contrast, fibril-exposed H4/WTS cells showed the formation of aggregates (Fig. 2g, bottom right, arrows). Since exogenous aSyn was only detected in naïve H4 cells as tiny dot-like structures at this exposure condition (0.1 µM fibrils, 1 h) (Fig. 2g, top right), the increase in large aggregates in H4/WTS was mainly attributed to the aggregation of endogenous aSyn. In a complementary experiment, we applied preformed histidine-tagged aSyn (His-aSyn) fibrils to H4/WTS cells, in order to distinguish exogenous aSyn (His-aSyn) from untagged endogenous aSyn based on their molecular weight differences on SDS-PAGE (Fig. 2h). In addition to monomeric aSyn (I for untagged-aSyn monomers and II for His-aSyn monomers), both His-tagged and untagged fibrils were characterized *in vitro* by the presence of  a particular oligomeric species  on SDS-PAGE, with a molecular weight of <58 kDa. Importantly, the molecular weight differed between untagged and His-tagged aSyn oligomers (III for untagged aSyn  oligomers, IV for His-aSyn  oligomers)  (Fig. 2h, *in vitro*). Interestingly, we detected both oligomeric species (III for endogenous unlabeled aSyn and IV for exogenous His-aSyn) in His-aSyn fibril-exposed H4/WTS cells, which were not detectable in untreated cells (Ctrl) (Fig. 2h, H4/WTS). In addition, a higher molecular weight smear was observed (Fig. 2h, blue frame), indicating an increase in the aggregation of endogenous aSyn.

To further validate that exogenous aSyn could trigger aggregation of endogenous aSyn, we studied the formation of soluble aSyn oligomers, the initial step of aSyn aggregation process, by using SEC (Fig. 2i). Subcellular distribution analysis in H4/WTS cells showed that endogenous aSyn is mainly soluble and localized within the cytosol (Supplementary Fig. S4c). For SEC analysis, we exposed H4 and H4/WTS cells to different aSyn species (1 µM for 6 h), performed biochemical fractionation, and loaded soluble cytosolic proteins on a SEC column, followed by a dot blot analysis of the resulting SEC fractions at given retention times. aSyn administration to both H4 and H4/WTS cells resulted in an overall increase in total aSyn signal in comparison to untreated cells (Ctrl) (Fig. 2i). As shown in Supplementary Fig. S5a, monomeric aSyn eluted at retention times (tR) of 19–22 min. In H4 cells treated with aggregated aSyn (oligomers and fibrils), an increase in aSyn signals was not clearly detectable in small oligomer-enriched fractions (Fig. 2i, top panel, tR: 17–18 min). However, in the presence of a high level of endogenous aSyn (Fig. 2i, H4/WTS, bottom panel), administration of oligomeric and fibrillar aSyn significantly shifted intracellular aSyn (including exogenous and endogenous aSyn) from monomers (tR: 19–22 min) toward oligomers (tR: 17–18 min). Since soluble exogenous aSyn was mainly detected in fractions of tR of 20–21 min derived from exposed H4 cells for 1 h, the increase in oligomer levels (tR: 17–18 min) referred mostly to endogenous aSyn in H4/WTS cells.

In summary, our complementary analyses demonstrated that extracellular aggregated aSyn species reach the cytosol of recipient cells and are capable of triggering the aggregation of endogenous aSyn.

### Exogenous aSyn is primarily localized along endosomal compartments, but not within lysosomes

**Figure 3:**
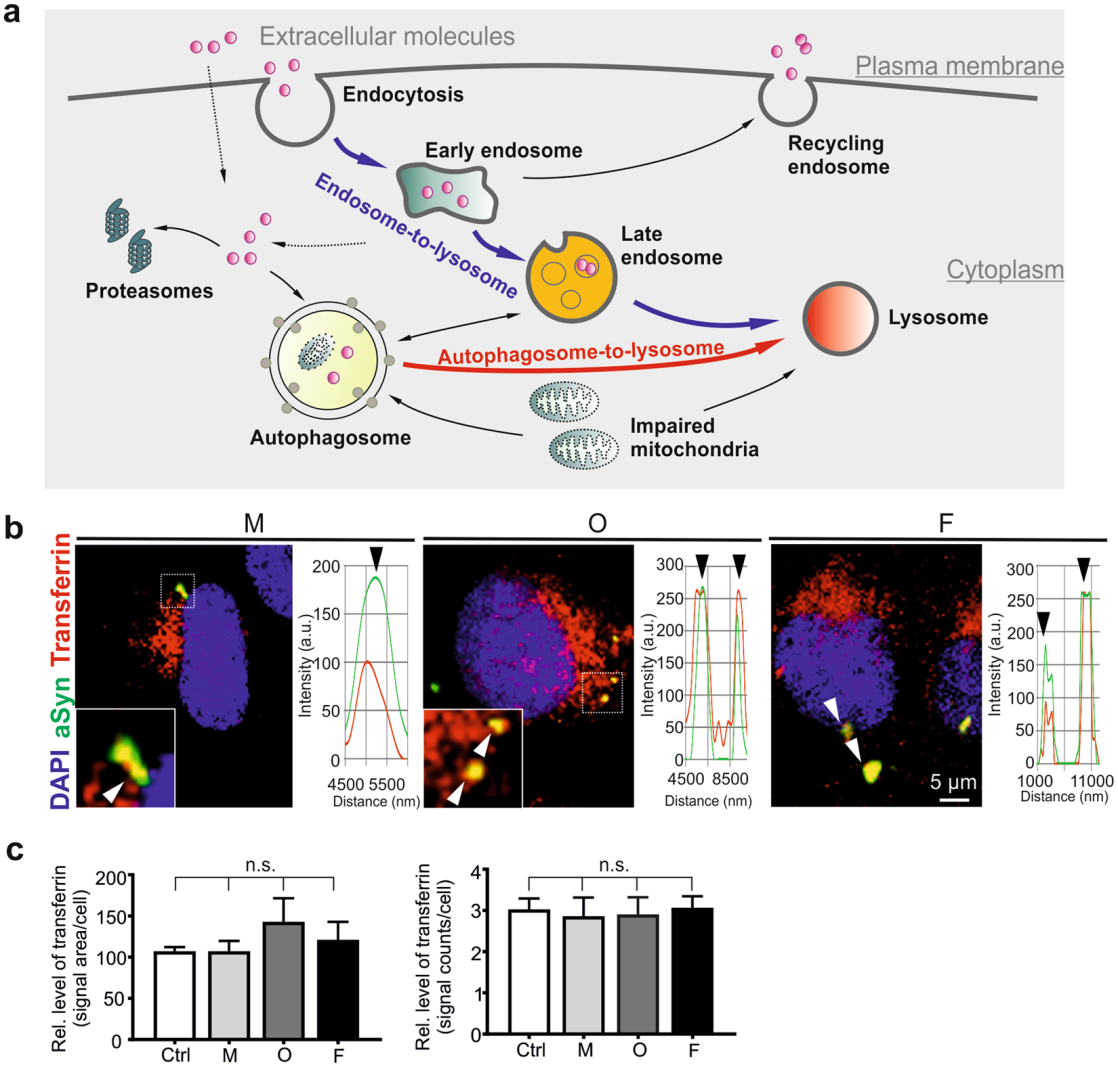
Uptake of extracellular aSyn by clathrin-mediated endocytosis. **(a)** Schematic diagram of trafficking and processing of extracellular molecules after internalization. (**b)** H4 cells were exposed to AlexaFluor568-labeled transferrin, followed by the administration of monomeric (M), oligomeric (O), or fibrillar (F) aSyn. Co-localization of aSyn (green) and transferrin (red) was analyzed by ICC and confocal microscopy. Enlarged views of selected regions (dashed frames) are shown at the bottom left corner of the images. Examples of co-localized signals are highlighted by arrowheads and confirmed by profile intensity plots. (**c)** Analysis of endocytosed transferrin levels, by determining either transferrin signal area/cell (left) or the counts of transferrin signal/cell (right), does not reveal significant differences between cells exposed to different aSyn species (n = 3, One-way ANOVA with Dunnett’s multiple comparisons test).

The endocytic pathway is made up of a dynamic vesicular network (Fig. 3a), initiated by the engulfment of extracellular materials into early endosomes. As major sorting stations, early endosomes either fuse with recycling endosomes to bring internalized materials back to extracellular environment, or mature into late endosomes for degradation in lysosomes^20^. Alternatively to degradation, endocytosed materials escape from endocytic compartments and reach the cytosolic space of the cell^21^. The endocytic pathway is closely interconnected with the ALP, via which misfolded/dysfunctional proteins and impaired organelles (e.g. mitochondria) are incorporated into autophagosomes, which utterly fuse with lysosomes, leading to decomposition and degradation of autophagosomal contents. In addition, autophagosomes can also fuse with late endosomes prior to being incorporated into lysosomes^20^.

To understand intracellular trafficking and functional consequences of exogenous aSyn, we investigated the localization of aSyn in distinct intracellular compartments upon uptake. Clathrin-mediated endocytosis is the major route of endocytic pathways and previous studies have suggested that extracellular aSyn can be internalized by dynamin-dependent clathrin-mediated endocytosis^22–24^. As the uptake of extracellular transferrin is well known to occur via clathrin-mediated endocytosis^25^, we analyzed the co-localization of aSyn with AlexaFluor568-labeled transferrin, which was applied to H4 cells 30 min prior to extracellular aSyn challenge. All forms of extracellular aSyn were co-localized, at least partially, with transferrin (Fig. 3b), supporting the involvement of clathrin-mediated endocytosis in aSyn uptake. Of note, analysis of transferrin immunosignals demonstrated that the endocytosis of transferrin *per se* was not significantly influenced by aSyn administration (Fig. 3c), suggesting that the endocytic pathways, in particular the clathrin-mediated endocytosis, are not impaired by exogenous aSyn, even by its highly aggregated forms.

**Figure 4:**
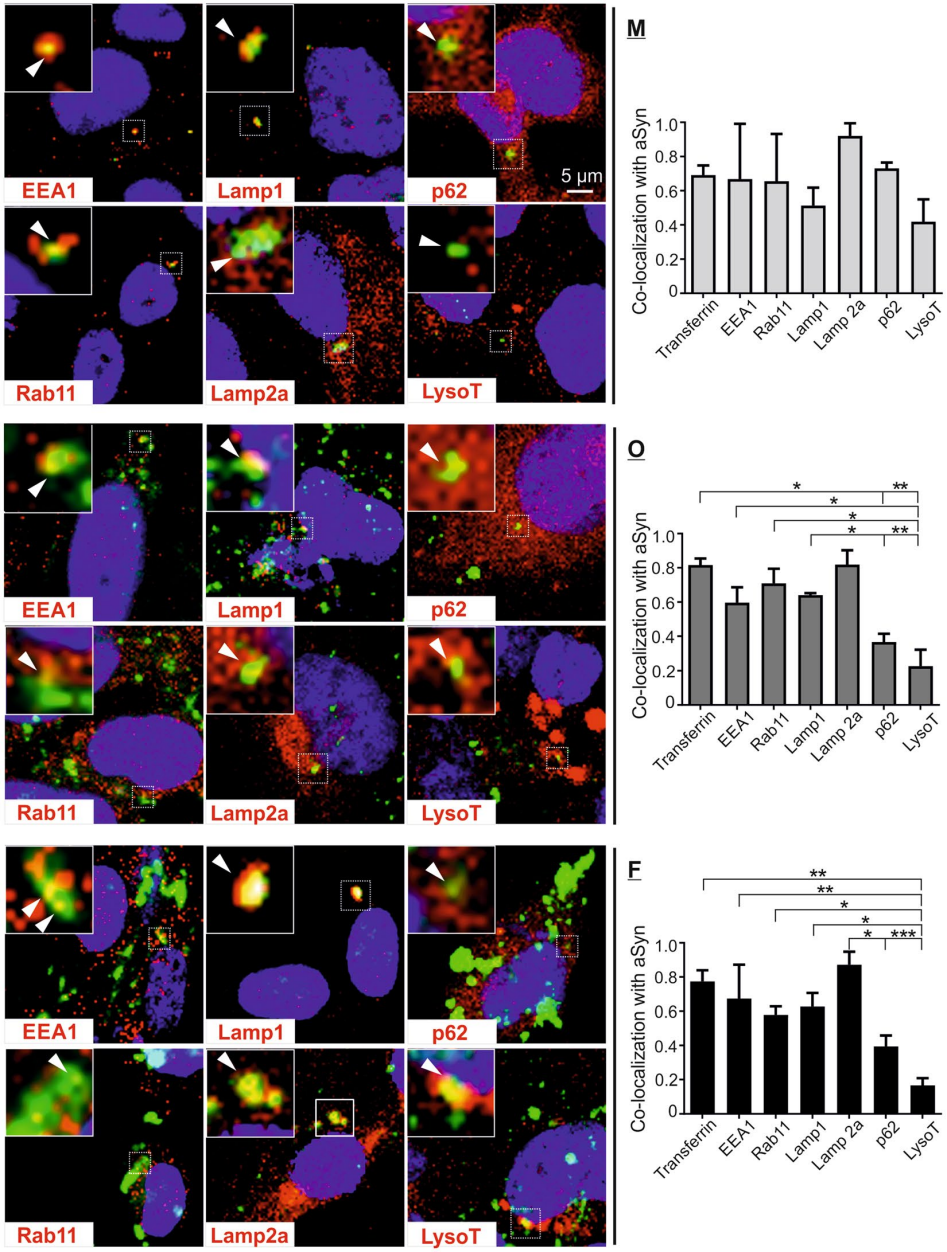
Co-localization of internalized aSyn species with markers of endosomal/lysosomal pathway. H4 cells were exposed to monomeric (M), oligomeric (O), and fibrillar (F) aSyn. Co-localization of exogenous aSyn (green) with markers of endosomal/lysosomal pathway (red), was analyzed by ICC and confocal microscopy. Analyzed markers included: EEA1 for early endosomes, Rab11 for recycling endosomes, Lamp1/Lamp2a for late endosomes and lysosomal membrane protein; p62 for autophagosomes and lysosomes, and LysoT for lysosomes. A selected region in each image is marked by a dashed frame and its enlarged view is shown at the top left corner of each image. Co-localization of aSyn and endosomal/lysosomal makers (yellow) in selected regions is highlighted by arrowheads and confirmed by the profile intensity plots (Supplementary Fig. S6). Quantification of the degree of co-localization between exogenous aSyn and endosomal/lysosomal markers as well as transferrin (right) demonstrates a generally  lower co-localization degree of aggregated aSyn with p62 and LysoT as compared to the co-localization of aSyn with other markers (n = 3, One-way ANOVA with Sidak’s multiple comparisons test).

In order to identify potential dysfunctions along endosome-to-lysosome and autophagosome-to-lysosome routes, we analyzed the co-localization of exogenous aSyn with markers of the endosomal-lysosomal pathway and the ALP (Fig. 4 left panel, Supplementary Fig. S6). Exogenous aSyn was co-localized with markers of early and recycling endosomes (EEA1 and Rab11, respectively), as well as with those of late endosomal and lysosomal membranes (Lamp1 and Lamp2a), independent of the initial aggregation state of extracellular aSyn. Furthermore, exogenous aSyn showed partial co-localization with the autophagosome/lysosome marker p62 and the lysosomal compartment visualized by using the acidotropic dye LysotrackerRed (LysoT). Notably, quantification revealed lower degree of co-localization between exogenous aSyn and p62, and especially LysoT than with other endosomal/lysosomal markers in oligomer- or fibril-exposed cells (Fig. 4 right panel).

### Extracellular aggregated aSyn alters lysosomal morphology and function

**Figure 5:**
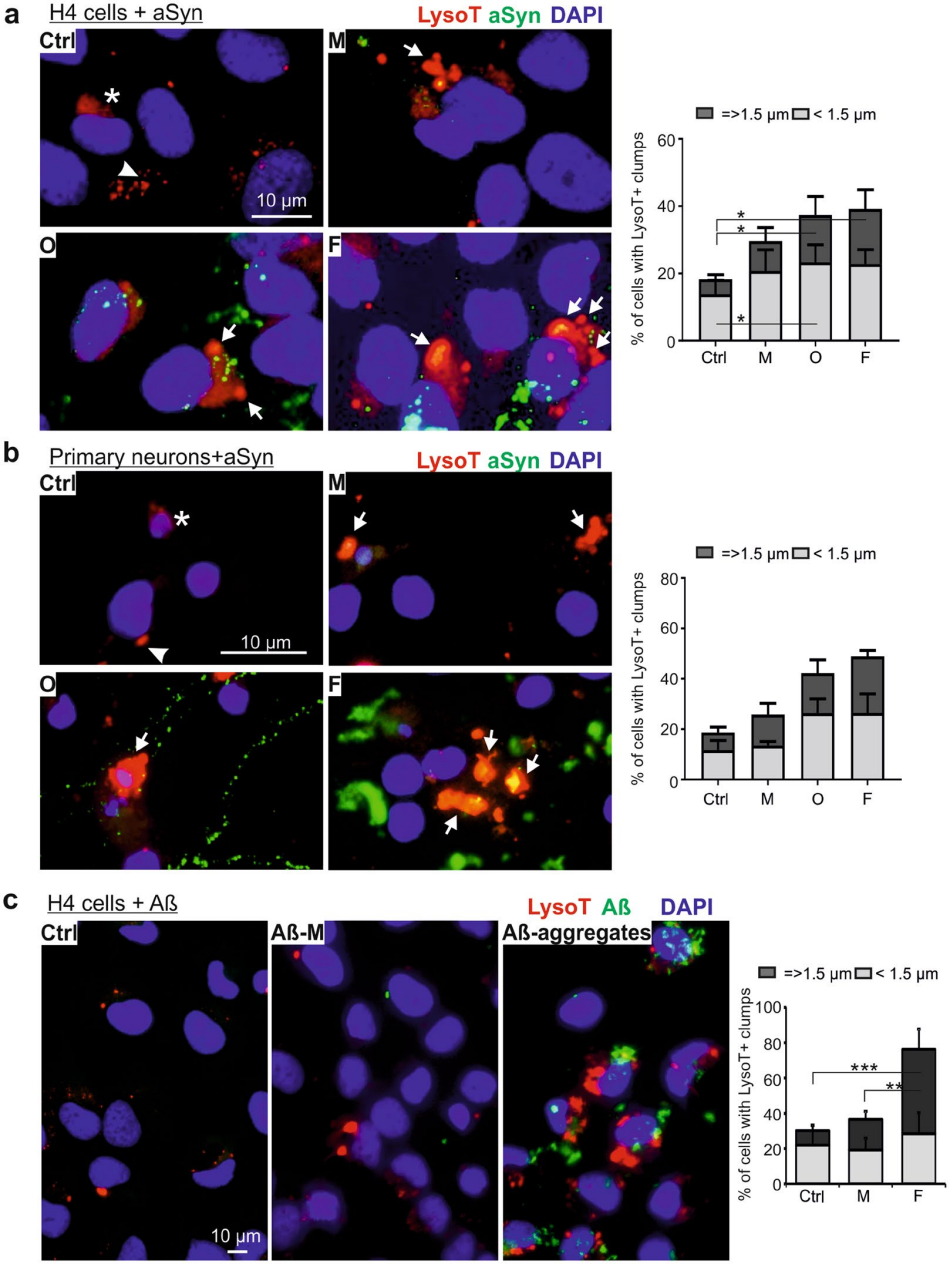
Morphological changes in lysosomes of aSyn or Aβ-exposed cells. **(a**,**b)** H4 cells (**a**) and rat primary neurons (**b**) were exposed to monomeric (M), oligomeric (O), and fibrillar (F) aSyn. Lysosomes were probed with LysoT and their morphology was analyzed by fluorescent microscopy (left panels of (**a**,**b**)). In untreated cells (Ctrl), LysoT staining demonstrates diffuse (asterisks) or small puncta-like structures (arrowheads). In both H4 cells and primary neurons, extracellular administration of aSyn induces a pronounced enlargement of lysosomes characterized by the formation of LysoT+ clumps (arrows). The proportion of cells carrying larger LysoT+ clumps (diameters = >1.5 µm) enhances in aSyn-exposed cells with increasing aggregation states of extracellularly applied aSyn. (**c)** Enlargement of lysosomes probed by LysoT (red) in H4 cells treated with 1 µM Aβ monomers (Aβ-M) and aggregates for 24 h (ICC: left; quantification: right). Quantification for (**a**–**c**), n = 3, Two-way ANOVA with Tukey’s multiple comparisons test.

Due to the substantial decrease in co-localization of exogenous aSyn with p62 and LysoT, we addressed the effects of exogenous aSyn on lysosomes. In untreated H4 cells, LysoT-labeled lysosomes were characterized by diffuse or small puncta-like structures (Fig. 5a). However, aSyn-exposed cells showed a pronounced accumulation of enlarged LysoT+ vesicles. Particularly, oligomer- and fibril-exposed cells demonstrated not only an increase in puncta-like structures but also a significant elevation in large lysosomal clumps. A similar trend was also observed in rat primary neurons upon aSyn exposure (Fig. 5b). To answer the question of whether the effect of aggregated aSyn on lysosomes is restricted to aSyn, amyloid beta 1–42 (Aβ) monomers and preformed aggregates were applied to H4 cells (Fig. 5c). We observed a pronounced accumulation of aggregated Aβ in recipient H4 cells. Moreover, Aβ aggregates significantly increased the accumulation of LysoT+ clumps, suggesting that the accumulation of dilated lysosomes may be a common effect of aggregating proteins related to neurodegenerative diseases.

**Figure 6:**
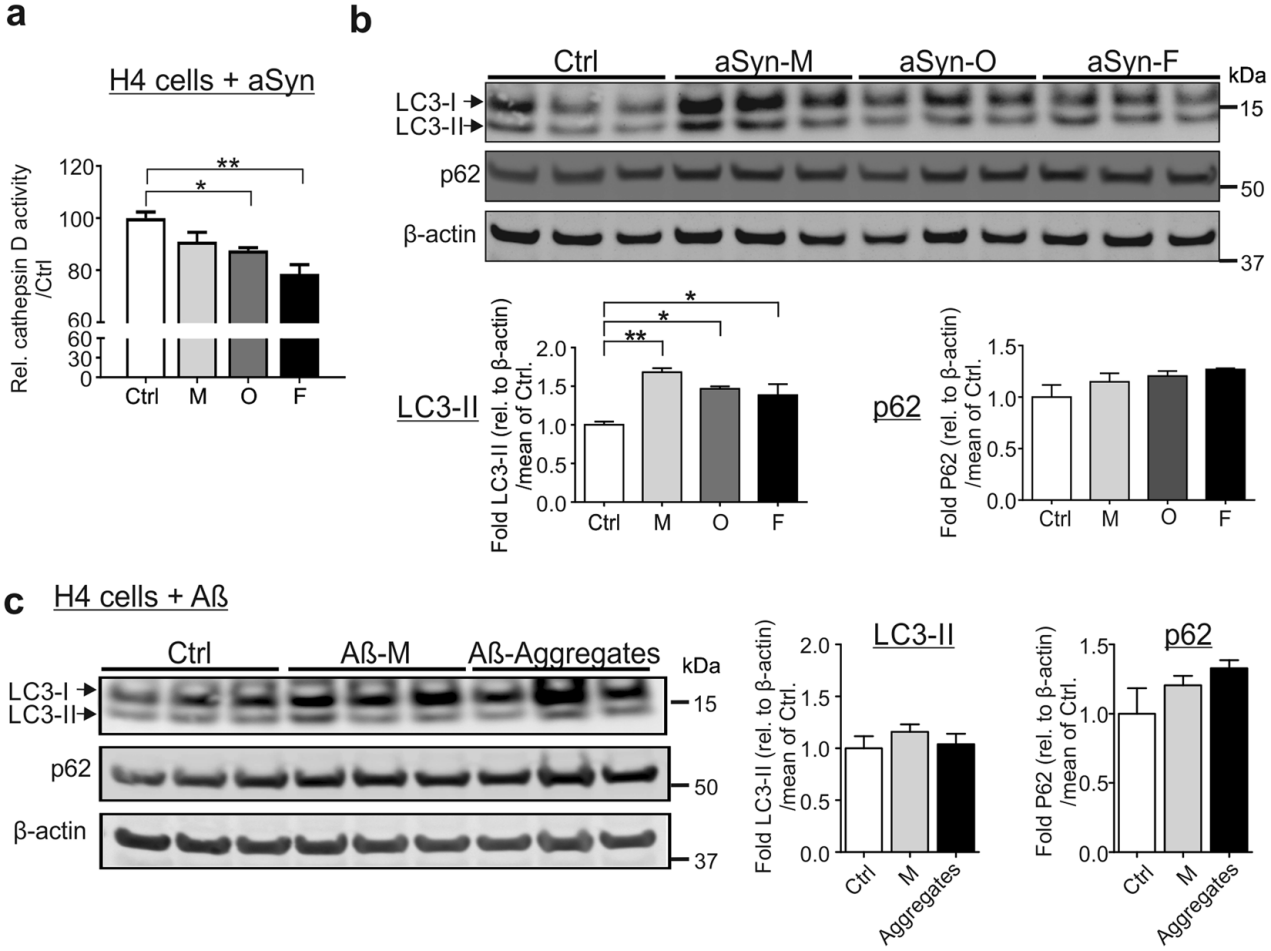
aSyn-mediated impairment in lysosomal activity and autophagosome turnover. **(a**,**b)** H4 cells were either untreated (Ctrl) or exposed to monomeric (M), oligomeric (O), and fibrillar (F) aSyn. **(a)** Lysosomal cathepsin D activity decreases significantly in oligomer- or fibril-exposed cells. (**b)** WB analysis of ALP markers LC3-II and p62 reveals a significant increase in LC3-II levels in aSyn-exposed cells and p62 up-regulation without reaching statistical significance. (**c)** H4 cells were either untreated (Ctrl), exposed to 1 µM Aβ monomers (Aβ-M), or Aβ aggregates. LC3-II levels do not change, whereas an up-regulation of p62 is measured without reaching statistical significance. Values are normalized to β-actin. WB bands for LC3-I/II, p62, and β-actin are derived from the same blot (quantification in (**a**,**b**), n = 3 and in (**c**), n = 4, One-way ANOVA with Tukey’s multiple comparisons tests).

To address whether enlarged lysosomes reflect alterations in their degradative potential, we treated H4 cells with the ALP inhibitor chloroquine. We observed that chloroquine induces a dose- and time-dependent formation of large lysosomal clumps in H4 cells as detected in aSyn-exposed cells (Supplementary Fig. S7). To confirm a functional impairment in lysosomes, we next determined the activity of the lysosomal enzyme cathepsin D. The assay was controlled by trehalose, an activator of ALP and by chloroquine (Supplementary Fig. S8). H4 cells exposed to oligomeric and fibrillar aSyn demonstrated a significant decrease in cathepsin D activity, indicating an impaired lysosomal activity (Fig. 6a). Overall our data supports that exposure to extracellular aSyn, in particular to aggregated aSyn, led to dilated, dysfunctional lysosomes.

We further examined whether the ALP activity (autophagosome turnover) is altered, using biochemical assessment of the autophagosome receptor protein p62 and the autophagosome membrane-conjugated form of LC3 (LC3-II) (Fig. 6b). While LC3-II levels indicate autophagosome formation, p62 levels reflect the efficiency of autophagosome degradation after fusion with lysosomes^26,27^. WB analysis revealed increased LC3-II levels, especially in monomeric, but also in aggregated aSyn-treated cells, suggesting a generally enhanced formation of autophagosomes upon aSyn administration. Levels of p62 were by approximately 20% increased in aSyn-exposed cells, suggesting an impaired autophagosome turnover, however the effect did not reach statistical significance. In parallel, we analyzed the ALP activity in H4 cells treated with Aβ monomers and aggregates (Fig. 6c). LC3-II did not change in response to the administration of Aβ monomers or aggregates. The mean p62 levels were increased especially for aggregates, however the effect did not reach statistical significance. In contrast to aSyn, none of the Aβ forms led to the accumulation of the autophagosome membrane-conjugated form of LC3 (LC3-II). Our data demonstrates that aSyn and Aβ aggregates comparably influence the accumulation of p62, suggesting a tendency to reduce the degradation of ALP substrates.

### Extracellular aSyn does not significantly change proteasome activity and mitochondrial homeostasis

**Figure 7:**
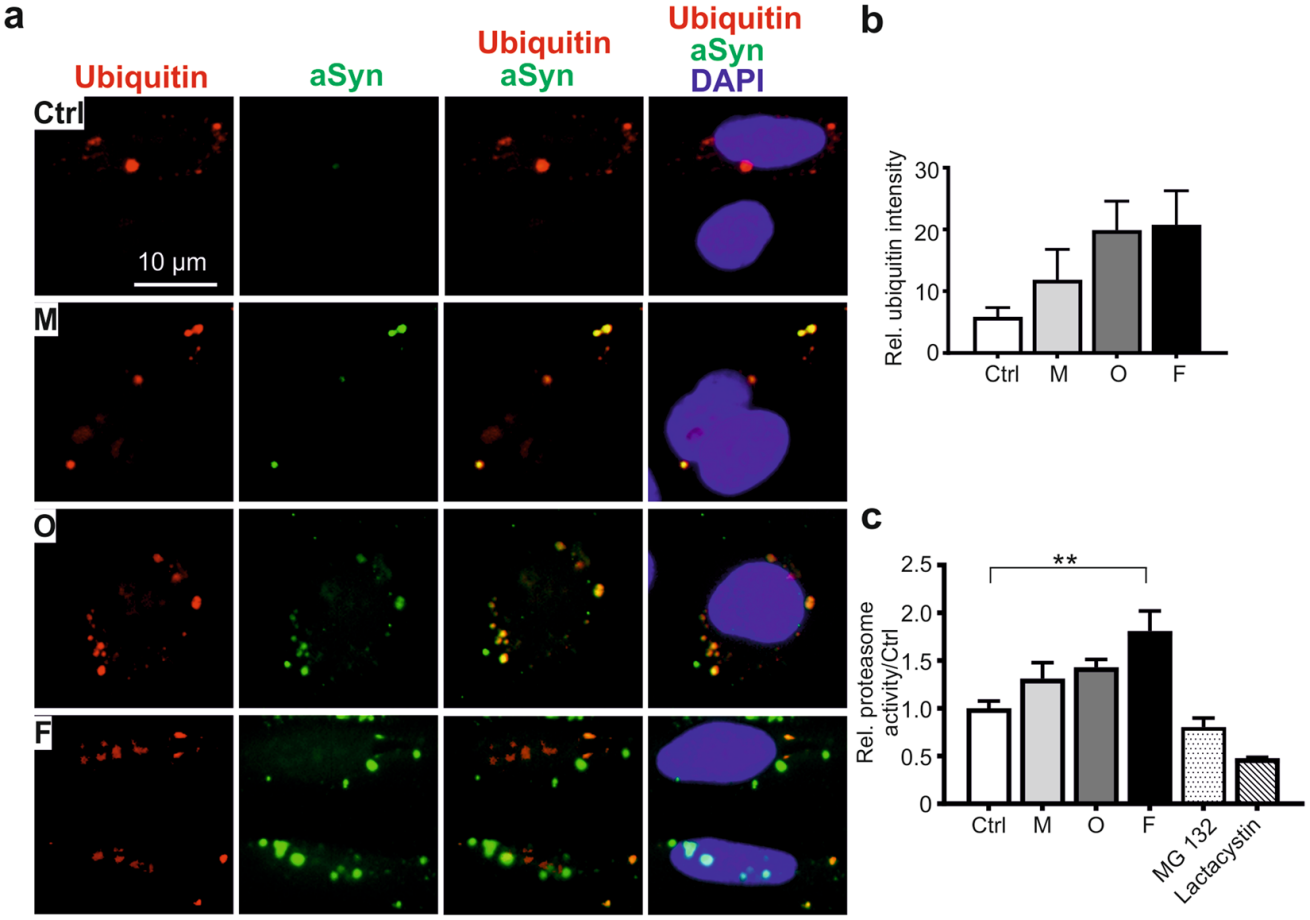
Effect of extracellular aSyn on the ubiquitin-proteasome system. H4 cells were exposed to monomeric (M), oligomeric (O), or fibrillar (F) aSyn. (**a)** ICC analysis of co-localization between exogenous aSyn (green) and ubiquitin (red). (**b)** Analysis of ubiquitin signal intensity as determined by the counts of cells with visible ubiquitin signals demonstrates a slight increase in ubiquitin expression in exposed cells. (**c**) Determination of proteasome activity in exposed cells shows an increased activity when compared to untreated cells (Ctrl) (quantification in (**b**,**c**), n = 3, One-way ANOVA).

In addition to the ALP, the ubiquitin-proteasome system is another important pathway for removing misfolded and aggregated proteins (Fig. 3a), and its dysfunction has been linked to PD pathology^28^. ICC analysis of ubiquitin in aSyn-exposed H4 cells showed that extracellular administration of aSyn does not reduce ubiquitin expression. In contrast, a trend towards increased ubiquitin immunosignals in aSyn-exposed cells was observed (Fig. 7a,b). Coincidently, extracellular aSyn, in particular the fibrillar aSyn species, induced a significant increase in proteasome activity (Fig. 7c).

**Figure 8:**
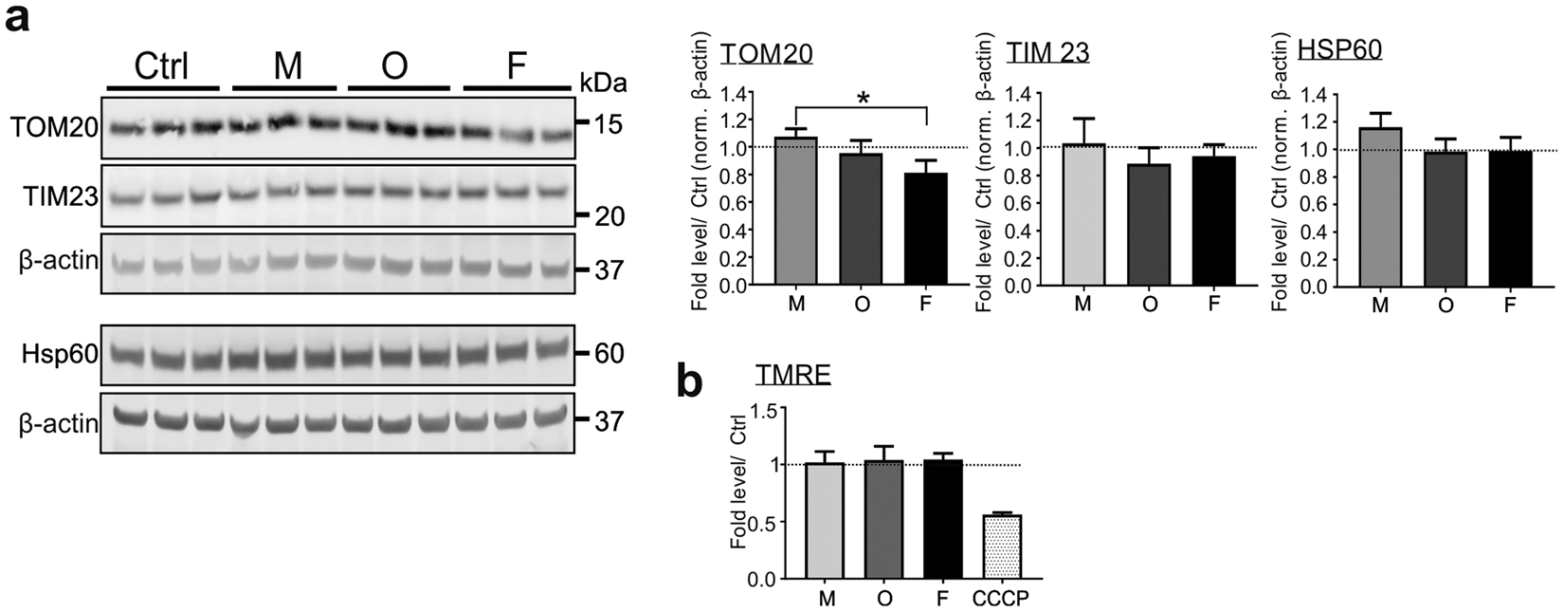
Impact of extracellular application of aSyn on mitochondria. Monomeric (M), oligomeric (O), or fibrillar (F) aSyn was applied to H4 cells. (**a)** WB analysis of the markers of mitochondrial mass (TOM20, TIM23, and HSP60). The bands for TOM20, TIM23, and β-actin (upper panel), as well as the bands for HSP60 and β-actin (lower panel) are derived from the same blots. Mitochondrial markers are normalized to β-actin of the identical blot. (**b)** Flow cytometric analysis of mitochondrial membrane potential-sensitive TMRE staining. Application of aSyn to cells does not change the mass of mitochondria and mitochondrial membrane potential, regardless of its aggregation state, when compared to untreated cells (Ctrl) (quantification in **(a**,**b**), n = 3, One-way ANOVA with Tukey’s multiple comparisons tests).

Disturbance of mitochondrial homeostasis, lysosomal dysfunction, and aSyn pathology are interconnected in PD pathology (Fig. 3a). To assess whether the extracellular aSyn-induced lysosomal phenotype is associated with mitochondrial damage, we analyzed mitochondrial mass by examining the levels of TOM20, TIM23, and HSP60, proteins located in the outer, the inner mitochondrial membrane, and in the mitochondrial matrix, respectively (Fig. 8a). We did not observe any significant differences between exposed and control cells, with the exception of TOM20 levels of cells exposed to fibrils, most likely due to a marginal increase in monomer-exposed, and a slight decrease in fibril-exposed cells. In total, these results suggest that the mitochondrial mass was not significantly affected by exogenous aSyn. Moreover, we did not detect an influence of exogenous aSyn on mitochondrial membrane potential, as measured via TMRE, a dye sensitive for changes in the mitochondrial membrane potential (Fig. 8b), supporting that mitochondrial activity was less affected.

### Trehalose efficiently reduces lysosomal alterations and the accumulation of exogenous aSyn

**Figure 9:**
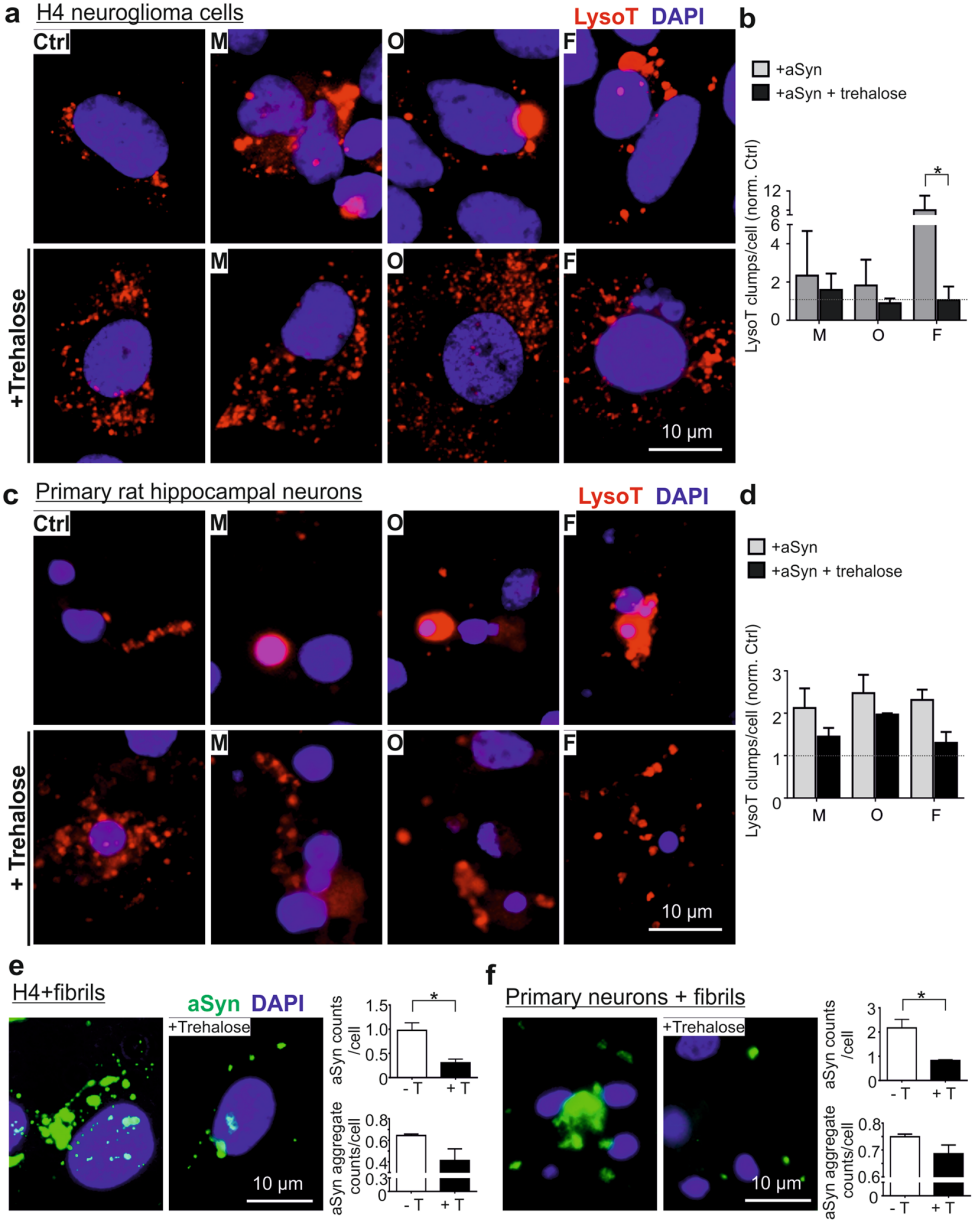
Effects of trehalose on exogenous aSyn-induced lysosome dilation and aSyn accumulation. H4 cells (**a**,**b**) and primary neurons (**c**,**d**) were treated with trehalose prior to aSyn application (M: monomers; O: oligomers; and F: fibrils). (**a**,**c)** ICC analysis of lysosomes probed by LysoT. (**b**,**d)** The amount of larger LysoT+ clumps induced by aSyn decreases with trehalose-pretreatment. Quantification was conducted by normalizing the levels in exposed cells against untreated cells (Ctrl) (n = 3, Two-way ANOVA with Sidak’s multiple comparisons test). (**e**,**f**) ICC analysis of extracellular aSyn accumulated in fibril-exposed H4 cells (**e**) and primary neurons (**f**) with or without trehalose (+T or -T) pretreatment. Quantitative analysis of ICC images reveals that trehalose decreases the accumulation of extracellular aSyn by determining the counts of detectable aSyn signals/cell or by assessing the counts of larger aSyn aggregates (area >2 µm^2^)/cell (n = 3, unpaired t-test).

We next investigated whether lysosomal and ALP activation prevents the deleterious effects of extracellular aggregated aSyn. We used trehalose, which is an mTOR (mechanistic target of rapamycin)-independent inducer of autophagy and enhances lysosomal biogenesis^29^. In H4 cells, application of trehalose alone increased lysosomal activity (Supplementary Fig. S8). We treated H4 cells with trehalose prior to aSyn exposure and observed that trehalose diminished the formation of large LysoT+ clumps (Fig. 9a,b), when compared to cells exposed to aSyn only. This effect was particularly significant in cells exposed to fibrils. As an accumulation of exogenous aSyn in fibril-exposed cells was readily detectable by ICC, we used this species for evaluating the effect of trehalose on the accumulation of exogenous aSyn. We observed that trehalose pretreatment reduces the accumulation of aSyn in exposed H4 cells (Fig. 9e), by determining either aSyn counts/cell or the counts of larger aSyn aggregates.

In the same set of experiments, we also tested the effect of rapamycin, an mTOR-dependent inducer of autophagy. Like trehalose, rapamycin also generally reduced the formation of abnormal LysoT+ clumps (Supplementary Fig. S9a) and the accumulation of exogenous aSyn (Supplementary Fig. S9b, left). However, in contrast to trehalose that preferentially prevented the formation of larger aSyn aggregates (Fig. 9e), rapamycin even induced a slight increase in larger aSyn aggregates in fibril-exposed H4 cells (Supplementary Fig. S9b, right). Therefore, trehalose was more efficient in promoting the clearance of large aSyn aggregates than rapamycin.

Importantly, the positive effects of trehalose in the reduction of dilated lysosomes and accumulation of exogenous aSyn, especially in the formation of larger aSyn aggregates, were also observed in primary neurons (Fig. 9c,d,f). Taken together, the findings from both H4 cells and primary neurons substantiate the protective effects of trehalose on aSyn mediated-impairment of lysosomes and on the clearance of accumulated exogenous aSyn in the recipient cells.

## Discussion

Cell-to-cell spreading of pathogenic aSyn has been implicated in PD and other alpha synucleinopathies by postmortem studies and by findings in a variety of cell and animal models. Non-motor symptoms of PD, including disturbances in sensory and autonomic function, may even occur more than 10 years before the onset of motor symptoms^30^. Such features can be speculated to be associated with aSyn deposition in olfactory bulb and enteric nervous system^10,31^. Therefore, it is important to understand mechanisms underlying aSyn aggregation along the routes of its propagation and to identify molecular and cellular targets for therapeutic prevention. In this study, we focused on the trafficking and accumulation of exogenous aSyn in recipient cells, the crucial steps in the propagation of aSyn pathology.

In order to identify aggregation-dependent alterations, we used label-free aSyn species (monomers, oligomers, and fibrils) because of two technical and biological considerations: First, aSyn labeling with tags may influence oligomerization/fibrillization of aSyn. For example, the widely used fluorescent tags are amine-reactive dyes that label lysine residues. However, manipulation of lysine residues in aSyn protein, either by modifications or by adding new lysine residues, may strongly impact the assembly of aSyn^19,32,33^. Second, monomers and preformed aggregated aSyn species may have variable reactivity to labeling reagents, leading to different labeling efficiency, which hinders a direct comparison. In order to follow exogenous, label-free human aSyn in recipient cells, we used 1) H4 cells, which express very low levels of endogenous aSyn, and 2) primary rodent neurons combined with using a human specific aSyn antibody (15G7). Both model systems allowed us to directly compare the behavior and consequences of extracellular aSyn in different aggregation states. Using AlexaFluor568 labeled and histidine-tagged aSyn, we validated the uptake and seeding feature of aggregated aSyn in recipient cells (Figs 1d and 2h). In line with a number of previous studies^16,23,34^, our results showed that monomeric aSyn, like other aggregated species, is internalized, although to a much less extent (Fig. 1a,b,e). The study of Lee *et al*. provided evidence that extracellular aSyn monomers can be internalized and then easily released by bi-directional diffusion across membrane^23^, suggesting that monomeric aSyn is internalized, probably by a different uptake mechanism from that for aggregated aSyn.

Using complementary analytical approaches, we showed that the administration of extracellular aggregated aSyn increases aSyn aggregation in recipient cells (Fig. 2). In particular, SEC analysis of oligomer- and fibril-exposed cells demonstrated an increased formation of cytosolic, soluble oligomers (Fig. 2i), the early aggregation intermediates of aSyn. Considering that the majority of endogenous aSyn of H4/WTS cells is localized in the cytosol as shown in the biochemical fractionation analysis of H4/WTS cells (Supplementary Fig.S4c), our results support that increased oligomerization of total intracellular aSyn in exposed cells is mainly attributed to endogenous aSyn via interaction with exogenous aSyn, which reaches the cytosol of recipient cells. Our previous study has shown that the oligomeric and fibril species used in this study exhibit toxicity to dopaminergic neurons^19^. Taken together, extracellular aggregated and neurotoxic aSyn species, but not monomeric species, are capable of triggering the aggregation of endogenous aSyn.

Previous data suggested that extracellular aSyn is internalized via dynamin-dependent, clathrin-mediated endocytosis. Concordantly, we also observed a co-localization of exogenous aSyn with transferrin (Figs 3b,c and 4), further supporting the involvement of clathrin-mediated endocytosis in aSyn uptake. Of note, the uptake of transferrin was not significantly affected by aSyn exposure, regardless of its aggregation states (Fig. 3c), indicating that endocytic activity is not impaired. Interestingly, extracellular aggregated aSyn species (oligomers and fibrils) were internalized more efficiently than monomeric aSyn, as revealed by their stronger accumulation in different cell types (Fig. 1a,b and Supplementary Fig.  S3) and their higher levels in the cytosol of recipient cells (Fig. 1e,g). In particular, aSyn fibrils maintained the aggregated feature at least for 24 h after uptake (Fig. 2e,f). Although the oligomers were less stable than fibrils in recipient cells, they exhibited a seeding effect in H4/WTS cells after 1 h treatment (Fig. 2i) and a small portion of oligomers was conserved after 24 h (Supplementary Fig. S5). In this study, we cannot exclude that exogenous aSyn, in particular the monomeric species, is released again into extracellular environment as suggested by Lee *et al*.^23^ and as supported by the co-localization of exogenous aSyn with Rab11 (Fig. 4). Nevertheless, our results indicate that aggregated aSyn accumulates more persistently than monomeric aSyn in recipient cells.

Tracking exogenous aSyn along the endosome-to-lysosome and autophagosome-to-lysosome routes, we observed a high degree of co-localization between aSyn and early, recycling, late endosomal markers (EEA 1, Rab11, and Lamp1), and the major lysosomal membrane associated proteins (Lamp1 and Lamp2a) (Fig. 4). These results are in accordance with the recent study of Domert *et al*.^16^, in which a significantly stronger co-localization of internalized aSyn with Lamp2a was described, when compared to markers of ER, multivesicular bodies, and synaptic vesicles. Interestingly, extracellular aggregated aSyn, but not monomers, showed a markedly weaker co-localization with p62 (ALP substrate) and LysoT. This finding suggests that exogenous aSyn, either in the endosome-to-lysosome or in the autophagosome-to-lysosome route, reaches lysosomes for its clearance but may not be internalized in lysosomes. Furthermore, the accumulation and enlargement of lysosomes were consistently observed in both H4 cells and primary neurons (Fig. 5). Lysosomal dilation concomitant with lysosomal dysfunction has been reported in some published studies^35,36^. Here, we provide novel evidence, linking altered lysosomal morphology with lysosomal impairment in the context of aSyn propagation: (1) Lysosomal activity decreased significantly upon the administration of aggregated aSyn (Fig. 6a); (2) Autophagosome turnover was compromised upon aSyn challenge, which is characterized by an increase in LC3-II levels and an accumulation of p62 (Fig. 6b). We also observed a similar response in p62 levels of Aβ aggregate-exposed cells. However, Aβ did not increase the accumulation of LC3-II, possibly reflecting that accumulation of autophagosomes is stronger upon exposure to aSyn than to Aβ aggregates (Fig. 6b,c); (3) Perturbed lysosomal activity is further substantiated by persistent accumulation of aggregated aSyn (Fig. 2); (4) The lysosomal phenotypes resemble those induced by chloroquine (Supplementary Fig. S7). Taken together, our results point towards an impaired lysosomal functionality in aSyn-exposed cells. This impairment is more pronounced in cells exposed to aggregated aSyn species.

The ALP and  the ubiquitin-proteasome system are major cellular degradation pathways, with a coordinate and complementary function in the maintenance of protein homeostasis^37^. We observed a slight increase in ubiquitin expression and in proteasome activity after aSyn exposure (Fig. 7), supporting a compensatory mechanism of the ubiquitin-proteasome system upon ALP impairment. Mitochondrial dysfunction, induced by e.g. aggregated aSyn^38–42^, can directly impair lysosomal activity, associated with the appearance of large lysosomal vacuoles^43^. Vice versa, defects of lysosomes have also been linked to mitochondrial dysfunction, potentially induced by an impaired lysosomal degradation of damaged mitochondria, reflected by altered mitochondrial mass^44^. Nevertheless, we did not observe changes in mitochondrial mass or mitochondrial membrane potential due to the extracellular administration of aSyn (Fig. 8). Overall, lysosomes as well as ALP appeared to be pronouncedly affected, especially by the aggregated aSyn species, while endocytic activity, the ubiquitin-proteasome system, and mitochondrial homeostasis of recipient cells were not significantly influenced by any forms of aSyn. Thus, data derived from our study, using identical cell models under the same experimental conditions, support that lysosomal impairment precedes disturbance of the ubiquitin-proteasome system and mitochondrial dysfunction, and consequently results in persistent accumulation of extracellular aggregated aSyn in recipient cells.

Although the observed detrimental effects on ALP and lysosomes induced by oligomers and fibrils were not significantly different between these species, the fibrils appeared to have a stronger effect than the oligomers. Emerging evidence from us^19,46^ and others^45^, indicates that the oligomeric aSyn species, or other aggregation intermediates, may represent the toxic species that contribute to neurodegeneration. Our results presented here suggest that internalized fibrils can also have strong harmful effects by overstressing the protein quality control systems. Furthermore, it is also possible that the fibrils are partly degraded by intracellular proteolytic systems into smaller toxic aggregated intermediates with seeding potential, potentiating the propagation of aSyn pathology.

In contrast to endogenous aSyn, exogenous aSyn is distributed in different compartments along its trafficking routes in recipient cells. It is still unclear whether and how the observed effects in ALP and lysosomes are triggered by a direct interaction of exogenous aSyn with ALP and lysosome-related compartments, which requires further studies.

Overall, the present study together with our previous finding that ALP is directly related to aSyn release^7,47^ strongly suggest a crucial role of lysosomes and ALP in the propagation of aSyn pathology. In line with disturbed lysosomes and ALP, we further showed that pretreatment with trehalose (Fig. 9) and rapamycin (Supplementary Fig. S9) reduces lysosome dilation and the accumulation of exogenous aggregated aSyn. In particular, trehalose was especially effective in reducing large aSyn aggregates. Trehalose, a disaccharide, has shown beneficial effects against the accumulation of neurotoxic, aggregated proteins and neurodegeneration^48^. A very recent study applying a lower dose of trehalose (25 mM) to aSyn fibril-exposed mouse primary neurons did not demonstrate significant effects in the clearance of aSyn fibril-induced aggregation and in the reduction of cell death^49^. This discrepancy to our study is probably due to dose-dependent effect of trehalose. Moreover, another recent study suggests that trehalose is able to suppress the pathogenic effects of aSyn in a rat model of PD, in particular in preventing behavioral and neurochemical defects as well as dopaminergic neurodegeneration^50^. Given the potential stability of trehalose in the brain because of low levels of trehalase^51^ and its safety for human consumption^52^, our results support a therapeutic potential of trehalose in preventing or halting the propagation of aSyn by targeting lysosomes. Namely, trehalose could be used as an orally-administered therapy by potentiating the clearance of aggregated aSyn, which could slow or reverse PD progression. Since dilated lysosomes were also observed in cells exposed to Aβ aggregates, we speculate that the beneficial effect of trehalose on preventing Aβ aggregation^53^ is attributed to its function in rescuing lysosomal dysfunction. Further studies are needed to identify more precise molecular lysosomal targets, validating the efficacy of trehalose as a therapeutic option for alpha synucleinopathies and other neurodegenerative diseases.

In summary, we demonstrate that the effect of extracellular aSyn strongly depends on the degree of its aggregation state. Extracellular aggregated aSyn is preferentially uptaken, more persistently accumulates within recipient cells, and more strongly impairs lysosomal activity and ALP, when compared to non-aggregated monomeric aSyn. Furthermore, perturbations in lysosomes and ALP are early events in response to extracellular aggregated aSyn (oligomers and fibrils), which are not only neurotoxic^19^, but also are capable of promoting the aggregation of endogenous aSyn. More importantly, administration of trehalose shows a promising therapeutic potential in preventing lysosomal alterations and the accumulation of aggregated aSyn.

## Methods

### Human aSyn and Aβ species

Preparation of human recombinant aSyn from *E*. *coli* as well as aSyn monomers, oligomers, and fibrils were performed as described previously^19,54^ with minor modifications. Briefly, monomers were purified from recombinant aSyn by using size exclusion chromatography (SEC). Oligomers were produced by incubating 70 µM aSyn monomers with 4 mM 4-hydroxy-2-nonenal (HNE, Cayman Chemical, Ann Arbor, MI, USA) in 30 mM Tris/HCl, pH 7.4 at 37 °C for 24 h. Fibrils were generated by agitating monomeric aSyn of 140 µM in 30 mM Tris/HCl pH 7.4 at 1000 rpm, 37 °C for 7 days. The formation of oligomers was controlled by SEC. Depending on preparation, about 60–80% monomers were converted to oligomers (an example SEC chromatogram is shown in Supplementary Fig. S5). The formation of fibrils was controlled by Thioflavin T, a dye that recognizes β-sheet structures and therefore binds to amyloid fibrils. Both oligomeric and fibrillar species have additionally been controlled either by electron microscopy or by atomic force microscopy^19,54^. The concentration of all aSyn species was examined by using BCA protein assay kit (Thermo Fisher Scientific, Rockford, U.S.A.).

Fibrils of N-terminal histidine-tagged recombinant aSyn (Sigma Aldrich, Munich Germany) were prepared by using the same protocol for unlabeled recombinant aSyn.

Labeling of aSyn monomers and preformed aSyn oligomers with AlexaFluro568 was performed with AlexaFluor®568 protein labeling kit according to the manufacturer’s instruction (Thermo Fisher Scientific, Waltham, MA, USA). Unreacted dye was removed by a fluorescent dye removal column (Thermo Fisher Scientific). The preparation of AlexaFluor568-labeled aSyn fibrils was carried out by agitating AlexaFluor568-labeled aSyn monomers using the standard protocol for unlabeled aSyn.

Human synthetic Aβ 1–42 peptide (1 mg lyophilized in TFA, Genicbio Limited, Shanghai, China) was dissolved in 200 µl PBS and 2 µl ammonia solution (25%). Aβ was diluted in PBS to a final concentration of 1 µg/µl. Aβ aggregates were prepared by constant agitation (750 rpm, 37 °C for 4 days). After agitation, the Aβ solution was centrifuged at 13000 g for 1 h at 4 °C. Aβ species concentration in the supernatant was measured (at 280 nm, molar extinction coefficient of 1474.041 L·mol^−1^·cm^−1^). The amount of insoluble Aβ aggregates in the pellet was estimated by subtracting the amount of Aβ in the supernatant from the starting amount of Aβ. Aβ aggregates were resuspended in PBS to a final concentration of 2 µg/µl and stored at –80 °C. The formation of Aβ fibrils were verified by Thioflavin T assay. For treatment, freshly prepared monomers were used.

### Cell culture and treatment

Naïve H4 human neuroglioma cells (ATCC, HTB-148) and a H4 cell line overexpressing wild type aSyn (H4/WTS)^32,55,56^ were maintained in Opti-MEM+ GlutaMAX (Invitrogen, Darmstadt, Germany) supplemented with 2 mM L-glutamine (Sigma Aldrich), 10% FCS (Sigma Aldrich), and 1% penicillin/streptomycin (Sigma Aldrich). For exposure experiment, cells were plated in 24-well plates (6 × 10^4^ cells/well) and 6-well plates (2.5 × 10^5^ cells/well), respectively.

Primary rat hippocampal neurons were prepared according to the protocol described previously^19^. Briefly, hippocampi were dissected from E18 Wistar rat embryos and dissociated with 0.25% trypsin (PAA Laboratories, Pasching, Austria) and 0.1 mg/ml DNase I (Roche Diagnostics GmbH, Penzberg, Germany). After washing with Neurobasal medium (Invitrogen), dissociated cells were resuspended in Neurobasal medium containing 2 mM L-glutamine and 1% B-27 supplement (Invitrogen) and plated in 24-well plates (4.6 × 10^4^ cells/well) or 6 cm dish (4 × 10^5^ cells/well). Animal procedures conducted were approved by the local Animal Welfare and Ethics committee of Erlangen, Germany and the animal care and use committee of the Friedrich-Alexander-University Erlangen-Nürnberg (TS-02/11) and all methods were performed in accordance with the relevant guidelines and regulations.

Exposure of H4 cells and primary neurons was performed 24 h and 7–14 days, respectively, after plating. If not given otherwise, cells were exposed to monomeric, oligomeric, and fibrillar aSyn at a concentration corresponding to 1 µM for monomeric aSyn for 24 h followed by washing with PBS twice. To modulate the degradation pathways, 100 mM trehalose (Tavarlin AG, Pfungstadt, Germany) or 20 nM rapamycin (Sigma-Aldrich) was applied to cells 30 min prior to aSyn treatment. To analyze clathrin-dependent endocytosis of transferrin, cells were incubated with 25 µg/ml transferrin AlexaFluor568 conjugate (Molecular Probes, Eugene, Oregon, USA) 30 min prior to aSyn exposure.

### Immunocytochemistry and fluorescence microscopy

For immunocytochemistry (ICC), cells grown on coverslips in 24-well plates were fixed with 4% paraformaldehyde (PFA) for 20 min. For staining, coverslips were incubated for 1 h in fish skin gelatin buffer (FSGB) with TritonX-100, followed by incubation with primary and secondary antibodies for 1 h, respectively. Subsequently, cells were incubated with 4′ 6′-diamidino-2-phenylindol (DAPI, Sigma-Aldrich, 1: 10000) for nuclear staining. Lysosomal staining of cultured cells was performed with LysotrackerRed (DND-99, Molecular Probes, 1:10000) for 30 min before fixation with 4% PFA. Thioflavin S staining was carried out by incubating the fixed cells with 0.5 mg/ml Thioflavin S (dissolved in water) for 5 min, followed by washing with 70% ethanol for 5 min prior to the staining with anti-aSyn antibody.

Fluorescence images were acquired by an Axiophot fluorescence microscope (Zeiss, Oberkochen, Germany) or one of following confocal microscopes: SP5 II Fast Resonant Scanner (Leica Microsystems, Wetzlar, Germany), LSM 780 confocal laser scanning microscope (Zeiss), and DMI 6000B inverted microscope equipped with a DFC 360FX camera (Leica).

Image processing was carried out by ZEN software (Carl Zeiss) as well as by Fiji^57^. Cell counting was performed with the Cell Counter of Fiji and morphological analysis of particles with Mito-Morphology Macro installed in Fiji^58,59^. The degree of co-localization was quantified by the determination of the thresholded Manderson’s overlap coefficient M2 by using the Colocalization Thresholds tool of Fiji. ICC images were counted using a systematic, random counting procedure. At least 3 images per slide were systematically selected at 40x magnification and counted after image acquisition. For each experiment, approximately 100–600 cells were analyzed.

### SDS-PAGE, Western blot, and dot blot

Cells were homogenized in PBS containing a protease inhibitor cocktail (Roche Diagnostics GMBH) with a glass potter homogenizer and protein concentration was determined by using BCA protein assay kit. For SDS-PAGE, protein samples were mixed with equal volume of 2X SDS sample buffer (0.125 M Tris/HCl pH 6.8, 4% SDS, 20% glycerol, 20 mM DTT, and 0.01% bromophenol blue), and 30–60 µg total protein was loaded on a 12% SDS-PAGE gel. For Western blot (WB) analysis of aSyn, the gel was blotted on a nitrocellulose membrane (Merk Millipore, Darmstadt, Germany). For dot blotting, a nitrocellulose membrane was placed in a dot blot apparatus (Minifold Dot-Blot System, Schleicher & Schuell, Dassel, Germany) and samples were loaded onto the membrane. For immunodetection of aSyn, blotted membranes were fixed for 30 min in 4% PFA to increase the sensitivity of aSyn detection^60^ and washed twice with TBS (50 mM Tris/HCl, pH 8.0, 150 mM NaCl). The membranes were next blocked in 3% bovine serum albumin (BSA) in TBS and probed with primary and horseradish peroxidase conjugated secondary antibodies. For visualization, chemiluminescent substrates (SuperSignal West Chemiluminescent Substrate kits, Thermo Fisher Scientific) were applied to the membranes, and chemiluminescent signals were detected by Gel Doc XR system (Bio-Rad Laboratories, Munich, Germany) and quantified by Image Lab Software (5.2.1, Bio-Rad). WB analysis of the ALP and mitochondrial markers was performed as described in our previous studies^47,56^.

### Blue Native PAGE

Blue native polyacrylamide gel  electrophoresis (BN-PAGE) was essentially performed according to Wittig *et al*.^61^. Cell lysates were prepared by homogenization in 25 mM Imidazole pH 7.5 containing 150 mM NaCl and 5 mM Ethylenediaminetetraacetic acid (EDTA) on ice. The cell lysate with 50 µg total protein, 500 ng aSyn monomer and preformed oligomers fibrils, as wells as standard proteins containing 10 µg alpha lactalbumin (14 kDa) and 10 µg phycoerythrin (250 kDa) were mixed with an equal volume of ice cold 85% sucrose in 25 mM Imidazole pH 7.5 containing 150 mM NaCl and 5 mM EDTA. The samples were then diluted with an equal volume of ice cold 1 M aminocaproic acid and shortly before electrophoresis, 1% Coomassie Brilliant Blue G stock solution (5% Coomassie Brilliant Blue G in 0.5 M aminocaproic acid) was added. Samples were separated by precooled 5–13% acrylamide gradient gels overlaid by a 3% stacking gel in the cold room. Gels were run at 100 V/15 mA with deep blue cathode buffer (50 mM Tricine. 7.5 mM Imidazole. 0.02% Coomassie G250) for the first third of the gel. Then the cathode buffer was exchanged to slightly blue cathode buffer (50 mM Tricine. 7.5 mM Imidazole. 0.002% Coomassie G250) and gels were run at 15 mA with unlimited voltage until the running front started to exit the gel. 25 mM imidazole/HCl pH 7.0 was used as anode buffer. Gels were blotted onto PVDF membranes by semi-dry blotting.

### Antibodies

The following primary antibodies were used for ICC and WB: Monoclonal mouse anti-aSyn Syn-1 (#610787, BD Biosciences, San Jose, U.S.A., WB: 1:2000), monoclonal rat anti-aSyn 15G7 (#ALX-804-258, Enzo Life Sciences AG, Lausen, Switzerland; ICC: 1:200), monoclonal mouse anti-human Aβ peptides (1E8, Bayer Schering Pharma AG, Berlin, Germany; ICC: 1:100), monoclonal mouse anti-βIII tubulin (#MAB3408, Chemicon International, Temecula, CA, USA, ICC 1:1000), polyclonal rabbit anti-β actin (#ab8227, Abcam, Cambridge, UK, WB: 1:2500), polyclonal rabbit anti-EEA1 (#ab2900, Abcam, ICC: 1:500), polyclonal rabbit-anti-GAPDH (FL-335) (#sc-25778, Santa Cruz Biotechnology, Dallas, Texas, USA, WB:1:1000), polyclonal goat anti-HSP60 (#sc-1052, Santa Cruz Biotechnology, WB: 1:1000), polyclonal rabbit anti-Lamp1 (#ab24170, Abcam, ICC: 1:50), polyclonal rabbit anti-Lamp 2a (#ab18528, Abcam, ICC: 1:250), polyclonal rabbit anti-MAP1LC3A, (#NB100-2331, Novus Biologicals, Littleton, Colorado, USA, WB:1:200), polyclonal rabbit anti-p62 (#PM045, MBL, Woburn, MA, USA, ICC: 1:100, WB: 1:500), monoclonal mouse anti-Rab11 (#610656, BD Transduction Laboratories, ICC: 1:100), monoclonal mouse anti-TIM23 (#611222, BD Biosciences GmbH, WB: 1:1000), polyclonal rabbit anti-TOM20 (#sc-11415, Santa Cruz Biotechnology, WB: 1:500), monoclonal mouse anti-ubiquitin (#sc-271289, Santa Cruz Biotechnology, ICC: 1:250). For immunodetection of aSyn, the Syn-1 antibody was used for Western and dot blot analysis, and the 15G7 antibody for ICC analysis.

The following secondary antibodies were used for ICC: AlexaFluor488 goat anti-rat, AlexaFluor568 goat anti-mouse, and AlexaFluor568 goat anti-rabbit (Life Technologies, 1:1000); and for WB: HRP goat anti-rabbit and anti-mouse IgG conjugates (Dianova, Hamburg, Germany, 1:10000).

### Biochemical fractionation

Cells were resuspended in buffer H (5 mM Tris/HCl, pH 7.4, with 300 mM sucrose, 0.1 mM EDTA, and protease inhibitor cocktail (Roche Diagnostics GmbH)) and homogenized with a teflon pestles for microcentrifuge tubes (VWR, Darmstadt, Germany). The total cell homogenate (T) was centrifuged at 1000 g, for 10 min at 4 °C (Supplementary Fig. S4a). After collecting the supernatant (S1), the pellet (P1) containing nuclei (Nu) was resuspended in buffer W (5 mM Tris/HCl, pH 7.4, with 0.1 mM EDTA, and protease inhibitor cocktail). The S1 fraction was further centrifuged at 17000 g for 30 min at 4 °C. The resulting supernatant (S2) with cytosolic proteins (Cyt) was saved, and the pellet (P2) was incubated in buffer W on ice for 30 min for hypo-osmotic lysis of intracellular vesicles and subsequently centrifuged at 17000 g for 30 min at 4 °C. The pellet (P3) containing cellular membrane (CM) was resuspended in 30 mM Tris/HCl buffer, pH 7.4. The supernatant (S3) was termed as remaining fraction (R). For analyzing the amount of aSyn in cytosolic and membrane fractions, 20 µg of total protein of T, Cyt, and CM fractions was loaded on SDS-PAGE. For analyzing subcellular distribution of aSyn, all fractions were adjusted to same volume by buffer H before loading on SDS-PAGE.

### Size exclusion chromatography

To analyze the formation of oligomers, 100–300 µg soluble cytosolic proteins (S1 fraction described in biochemical fractionation) were loaded on a Yarra 3 µ Sec-3000 column (Phenomenex, Aschaffenburg, Germany) using 50 mM Tris/HCl, pH 7.0, 150 mM NaCl as mobile phase with a flow rate of 0.5 ml/min. From 0–10 min, 5 fractions with 1 ml each were collected. From 10 min, one fraction of 0.5 ml per minute was collected. Afterward, collected fractions were analyzed by dot blot for immunodetection of aSyn.

### Flow cytometry

Flow cytometry was performed with three laser BD FACSCanto II (BD Biosciences) and BD FACSDiva 6 (BD Biosciences) analysis and quantification software. The mitochondrial membrane potential was analyzed by incubating 100 nM TMRE (tetramethylrhodamine, ethyl ester, Invitrogen) with cells in a 24-well plate at 37 °C for 20 min as described in detail in our previous study^61^. Cells were lifted with Accutase (Sigma Aldrich) and washed with PBS. The mean cellular fluorescence intensity of TMRE was analyzed by measuring 20000 single cells. As control, cells were treated with the mitochondrial oxidative phosphorylation uncoupling agent carbonyl cyanide m-chlorophenyl hydrazone (CCCP, 50 μM) for 10 min, followed by TMRE application.

### Cathepsin D activity

Cathepsin D activity was determined using the Cathepsin D activity assay Kit (Abcam). Cells plated in a 12-well plate with a density of 1.5 × 10^5^ cells/well were lysed in 200 µl chilled CD cell lysis buffer supplied in the kit. Cells and the reaction mix were prepared according to manufacturer’s protocol. The fluorescence derived from cathepsin D-mediated substrate cleavage was measured using a ClarioStar microplate reader (BMG LABTECH, Ortenberg, Germany) at excitation/emission = 328/460 nm.

### Proteasome activity

Cells in a 6-well plate were washed twice with prewarmed PBS and collected in 500 µl chilled PBS. Cell pellets were resuspended in 150 µl lysis buffer (10 mM Hepes, pH 7.9, 10 mM KCl, 1.5 mM MgCl_2_, and 1 mM DTT) and incubated for 30 min on ice followed by homogenization in a glass potter homogenizer. Cell homogenate was centrifuged at 14000 g for 10 s at 4 °C. The resulting supernatant was combined with 15 µl 300 mM Hepes buffer (pH 7.9, containing 1.4 M KCl, 30 mM MgCl_2_ and 1 mM DTT) and centrifuged at 14000 g at 4 °C for 70 min. The generated cytoplasmic supernatant was used for determination of proteasome activity by using the Proteasome Activity Assay Kit (Abcam) according to manufacturer’s protocol. The fluorescence produced by the cleavage of 7-amino-4-methylcoumarin (AMC)-labeled substrate was determined using a ClarioStar microplate reader at excitation/emission = 350/460 nm. As controls, proteasome inhibitors, MG132 (0.5 µM) or lactacystin (1 µl, supplied in kit), were either added to cells (MG132, 24 h) or to cytoplasmic supernatant (lactacystin).

### Statistical analysis

GraphPad Prism was used to perform statistical analyses (GraphPad Software). All numeric results are shown as mean ± standard error of the mean (SEM) and represent data from a minimum of three independent experiments. Analysis of significance was performed by using one-way ANOVA with Dunnett’s or Tukey’s comparisons test, two-way ANOVA with Tukey’s or Sidak’s multiple comparisons test, or unpaired t-test depending on the data sets. The respective tests used are specified in the corresponding figure legends. Significant differences are depicted as follows: *p < 0.05, **p < 0.01, ***p < 0.001, and ****p < 0.0001.

## Electronic supplementary material

Supplementary Information
