# Supplementary material for: Extracellular aggregated alpha synuclein primarily triggers lysosomal dysfunction in neural cells prevented by trehalose
Source: Sci Rep. 2019 Jan 24;9:544. doi: 10.1038/s41598-018-35811-8 (PMC6345801; doi:10.1038/s41598-018-35811-8)
Supplement: Supplementary file 1 — Supplementary Information [file 41598_2018_35811_MOESM1_ESM.pdf]

Extracellular aggregated alpha synuclein primarily triggers lysosomal dysfunction in neural cells prevented by trehalose

Anna-Carin Hoffmann[1], Georgia Minakaki[2], Stefanie Menges[2,3], Rachele Salvi[2], Sergey Savitskiy[1,4], Aida Kazman[1], Hugo Vicente Miranda[5], Dirk Mielenz[6], Jochen Klucken[2], Jürgen Winkler[2], Wei Xiang[1*]

[1]Institute of Biochemistry, Friedrich-Alexander-University Erlangen-Nürnberg (FAU), Erlangen, Germany

[2]Division of Molecular Neurology, University Hospital Erlangen, FAU Erlangen-Nürnberg, Erlangen, Germany

[3]Center for Human Genetics Regensburg, Regensburg, Germany

[4]Department of Chemistry, Technical University of Munich, Munich, Germany

[5]CEDOC, Chronic Diseases Research Center, NOVA Medical School | Faculdade de Ciências Médicas, Universidade NOVA de Lisboa, Campo dos Mártires da Pátria, 130, 1169-056 Lisboa, Portugal

[6]Division of Molecular Immunology, Nikolaus-Fiebiger-Center, Department of Internal Medicine III, FAU Erlangen-Nürnberg, Erlangen, Germany

E-mail addresses:

Anna-Carin Hoffmann: anna-carin.hoffmann@fau.de
Georgia Minakaki: Georgia.Minakaki@uk-erlangen.de
Stefanie Menges: Stefanie.Menges@uk-erlangen.de
Rachele Salvi: Rachele.Salvi@uk-erlangen.de
Sergey Savitskiy: s.savitskiy@tum.de
Aida Kazman: kazman.aida@googlemail.com
Hugo Vicente Miranda: hmvmiranda@nms.unl.pt
Dirk Mielenz: dirk.mielenz@fau.de
Jochen Klucken: Jochen.Klucken@uk-erlangen.de
Jürgen Winkler: Juergen.Winkler@uk-erlangen.de
Wei Xiang: wei.xiang@fau.de

[*]To whom correspondence should be addressed:

Wei Xiang, PhD:

Institute of Biochemistry, FAU Erlangen-Nürnberg, Fahrstraße 17, 91054 Erlangen, Germany, Tel.: +49-9131-8526206, Fax: +49-9131-8522485
E-mail: wei.xiang@fau.de

Supplementary information

Supplementary Fig. S1. Effect of extracellularly added aSyn on the viability of H4 cells.

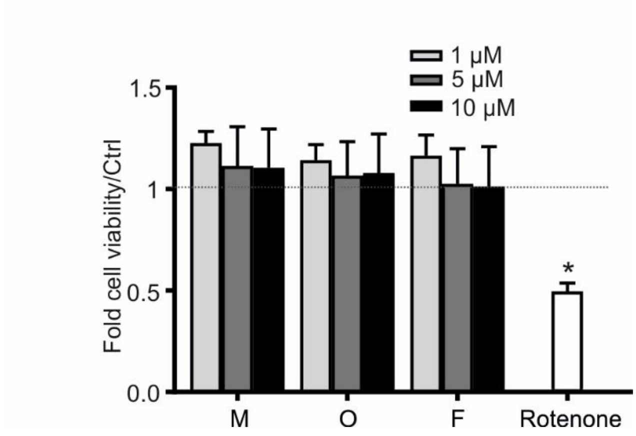

H4 cells were treated with monomeric (M), oligomeric (O), and fibirillar (F) aSyn of 1, 5, or 10 μM for 24 h. Cell viability was measured by using the CellTiter-Glo Luminescent Cell Viability Assay Kit (Promega). Administration of aSyn between 1-10 μM did not induce massive loss of H4 cells. Toxic agent rotenone of 5 μM was used as a control of the viability assay.

Supplementary Fig. S2. Quantitative PCR analysis of aSyn mRNA in H4 and H4/WTS cells exposed to extracellular aSyn.

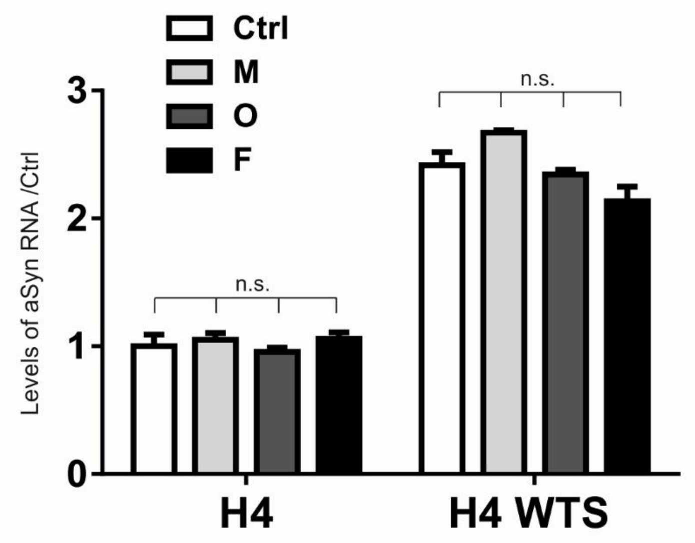

RNA isolation was performed by using the E.Z.N.A. Total RNA kit (Omega biotek store, Norcross, GA, USA) and reverse transcription by Superscript II plus (Life technologies). Quantitative PCR analysis was carried out by using specific primers for human aSyn and beta-actin genes (forward: aSyn, TTCAAGCCTTCTGCCTTTCCA; beta-actin, CTACGTGGCCCTGGACTTCGAGC) (reverse: aSyn, CTTCTCAGCCACTGTTGCCA; beta-actin, GATGGAGCCGCCGATCCACACGG) and a LightCycler 480 system (Roche). Melting curve analysis was used for quantification. The expression levels of aSyn gene were normalized to those of beta-actin. The results show that exposure of H4 and H4/WTS cells to extracellular monomeric (M), oligomeric (O), and fibrillar (F) aSyn does not significantly change the aSyn mRNA levels, as compared to untreated cells (Ctrl).

Supplementary Fig. S3. Accumulation of extracellularly added aSyn in differentiated LUHMES dopaminergic neurons and CG4 oligodendrocytes.

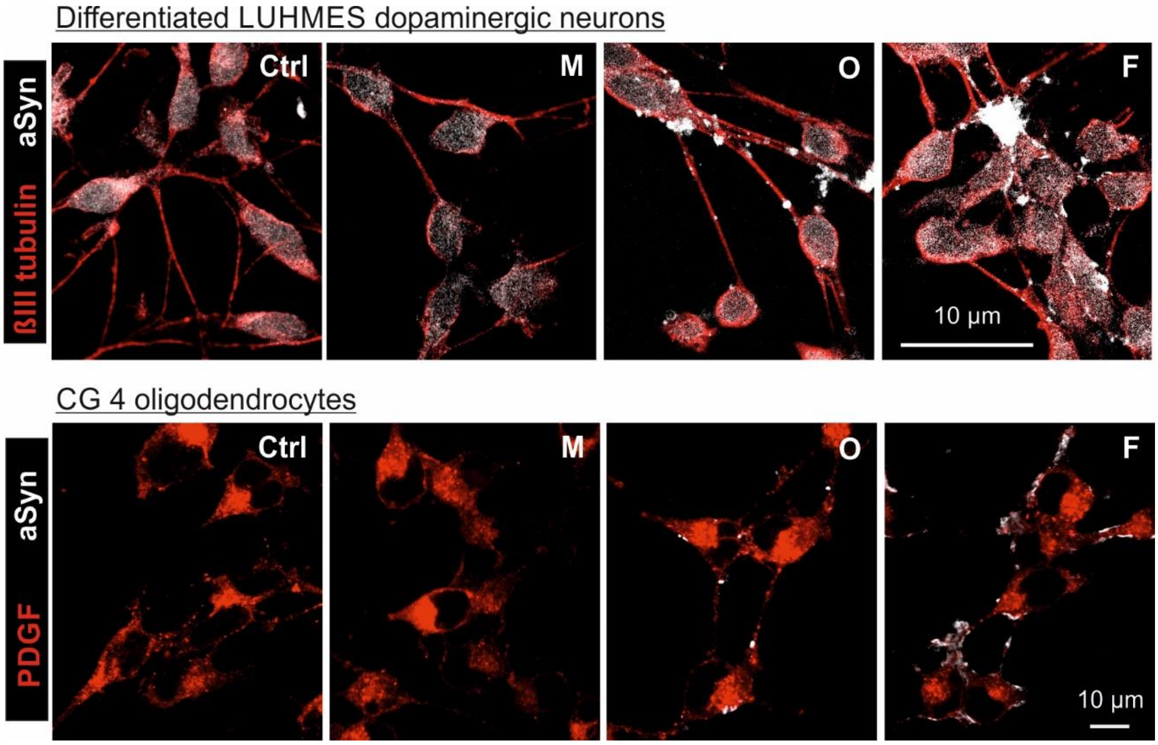

LUHMES cells were maintained in proliferation medium Advanced DMEM/F12 medium (Invitrogen) with 2 mM L-Glutamine (Sigma Aldrich), 1X N2 supplement (Invitrogen), 40 ng/ml FGF (Pepro Tech, Hamburg, Germany), 1% penicillin/streptomycin (Sigma Aldrich). All culture flasks and microtiterplates were pre-coated with 50 µg/ml poly-L-ornithine (Sigma-Aldrich) and 1 µg/ml fibronectin (Sigma Aldrich) at 37°C overnight. For cell differentiation, cells were grown in a T75 flask with a density of 4 x 10^4 cells/cm^2 in proliferation medium for 24 h. The medium was replaced at day 0 by differentiation medium Advanced DMEM/F12 medium with 2 mM L-Glutamine, 1 x N2 supplement, cAMP, tetracycline, human glial cell line derived neurotrophic factor (GDNF), and penicillin/streptomycin (Sigma Aldrich) until day 2. The predifferentiated cells on day 2 were then replated in a new pre-coated culture dish (density 1.4 x 10^5 cells/cm^2) for differentiation until day 6.

Rat oligodendrocytic progenitor CG4 cells were cultured in poly-L-ornithine coated cell culture flasks. Cells were grown in DMEM (PAN-Biotech, Aidenbach, Germany) supplemented with 4.5 g/l glucose, glutamine, N2 (Invitrogen), biotin (10 ng/mL), basic fibroblast growth factor (10 ng/mL), platelet derived growth factor (10 ng/mL), and in the presence of penicillin-streptomycin (10 ng/ml).

Cells were incubated either without aSyn (Ctrl) or with monomeric (M), oligomeric (O), and fibrillar (F) aSyn. Aggregated aSyn species (O and F) accumulate more strongly in differentiated LUHMES dopaminergic neurons (top panel, aSyn in white and βIII tubulin in red) and CG4 oligodendrocytes (bottom panel, aSyn in white and PDGF in red) than monomeric aSyn. PDGF was probed with polyclonal goat anti-PDGFRα antibody (#AF1062, R&D systems, Wiesbaden-Nordenstadt, Germany, dilution: 1:500).

Supplementary Fig. S4. Biochemical fractionation.

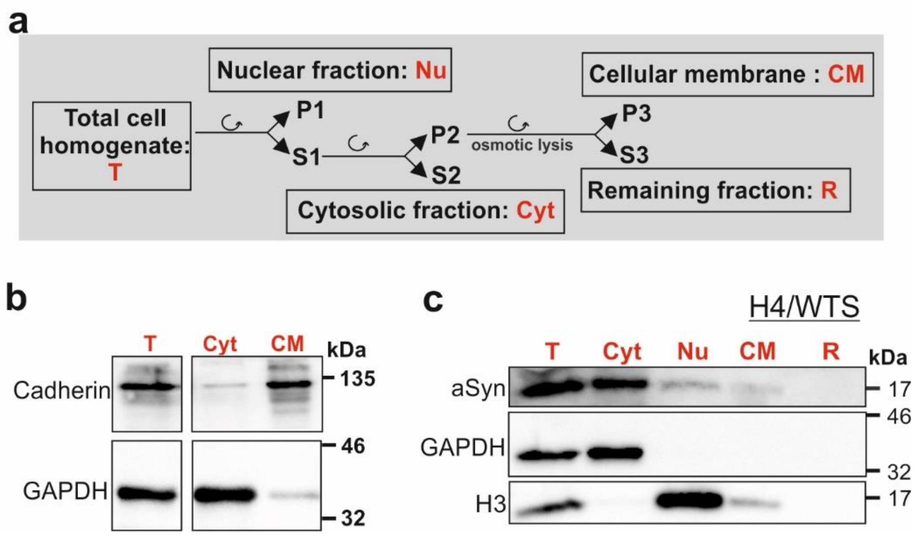

a) Schematic workflow. **b**) Extraction of cytosolic (Cyt) and membrane (CM) fractions is confirmed by the membrane marker cadherin and cytosolic marker GAPDH, respectively. **c**) Biochemical fractionation of H4/WTS cells and WB analysis of all fractions by anti-aSyn Syn1 antibody. The result demonstrates that endogenous aSyn is mainly localized in the cytosol. Extraction of nuclear fraction was confirmed by histone 3 (H3). Cadherin and histone 3 (H3) were probed by monoclonal mouse anti-pan-Cadherin (CH-19) (#ab6528, Abcam, Cambridge, UK, WB: 1:1000) and monoclonal rabbit anti-Histone H3 (D1H2) XP (#4499, Cell Signaling Technology, Cambridge, UK, WB: 1:2000), respectively.

Supplementary Fig. S5. SEC analysis.

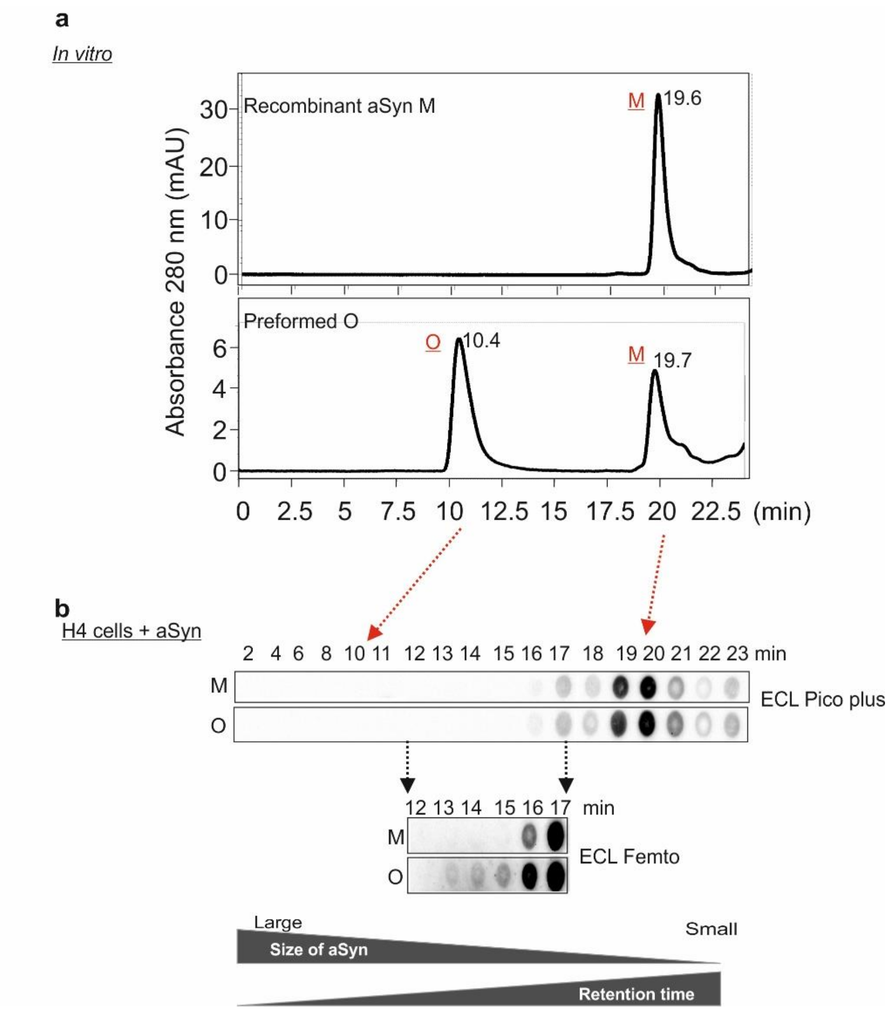

a). SEC analysis of human recombinant aSyn monomers and preformed aSyn oligomers. Absorbance at 280 nm shows that aSyn monomers elute at a retention time (tR) of ~ 19.6 min. Preformed oligomers contains aSyn monomers (tR~ 19.7 min) and aSyn oligomers (tR: ~ 10.4 min). The preformed oligomer preparation contained approximately 65% oligomers in this example chromatogram. **b).** SEC and dot blot analysis of cytosolic soluble fractions of H4 cells exposed to 1 µM aSyn monomers (M) or oligomers (O) for 24 h. Using the ECL femto detection kit (Thermo Fisher Scientific), oligomers in the fractions collected after retention times of 12-15 min are visible in oligomer-exposed cells.

Supplementary Fig. S6. Profile intensity plots of co-localization of exogenous aSyn with endosomal/lysosomal markers.

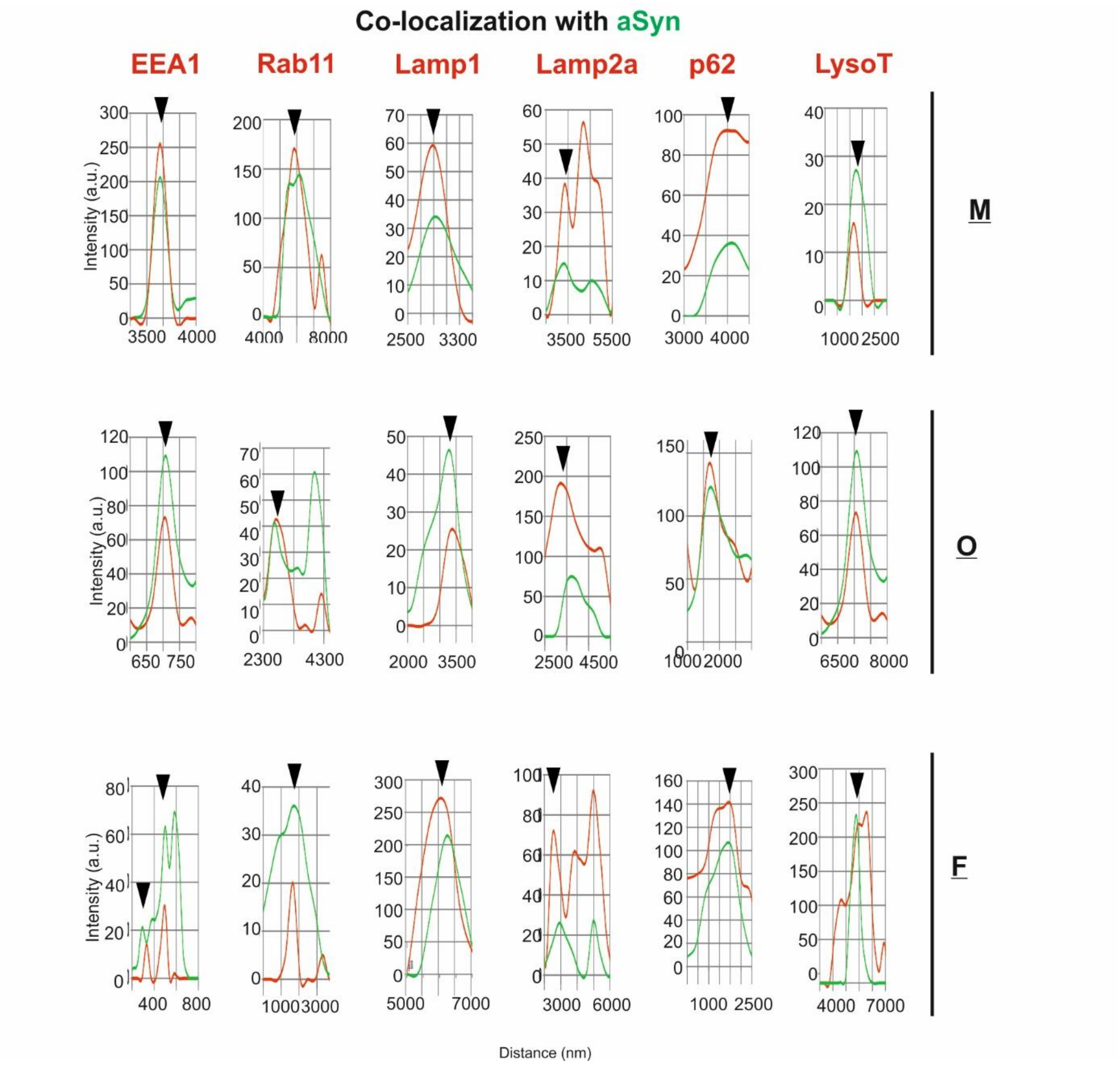

Profile intensity plots confirm the co-localization of exogenous aSyn with endosomal/lysosomal markers shown in Fig. 4. Black arrow heads correspond to white arrow heads in Fig. 4.

Supplementary Fig. S7. Induction of lysosome dilation by chloroquine.

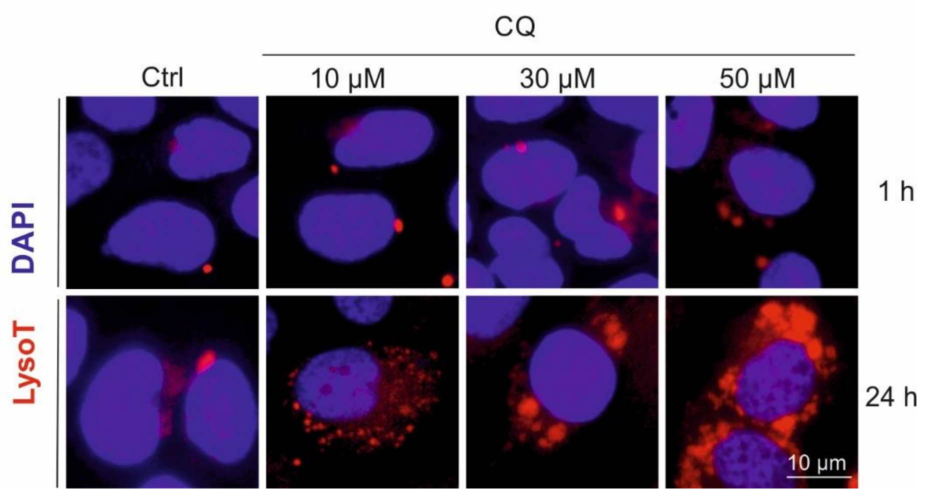

H4 cells were treated with 10 - 50 μM chloroquine (CQ) at 37°C for 1 - 24 h and subsequently stained with LysoT.

Supplementary Fig. S8. Control of cathepsin D assay.

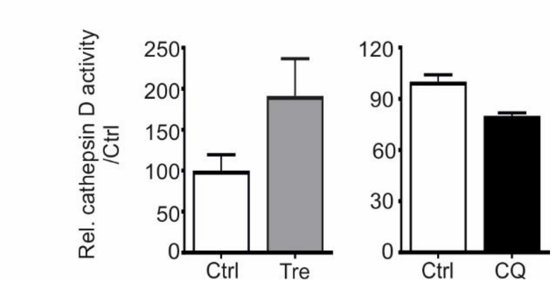

Cathepsin D activity assay was controlled by using trehalose (Tre), an activator of ALP, and chloroquine (CQ), an inhibitor of ALP.

Supplementary Fig. S9. Effects of rapamycin on extracellular aSyn-induced lysosome dilation and aSyn accumulation.

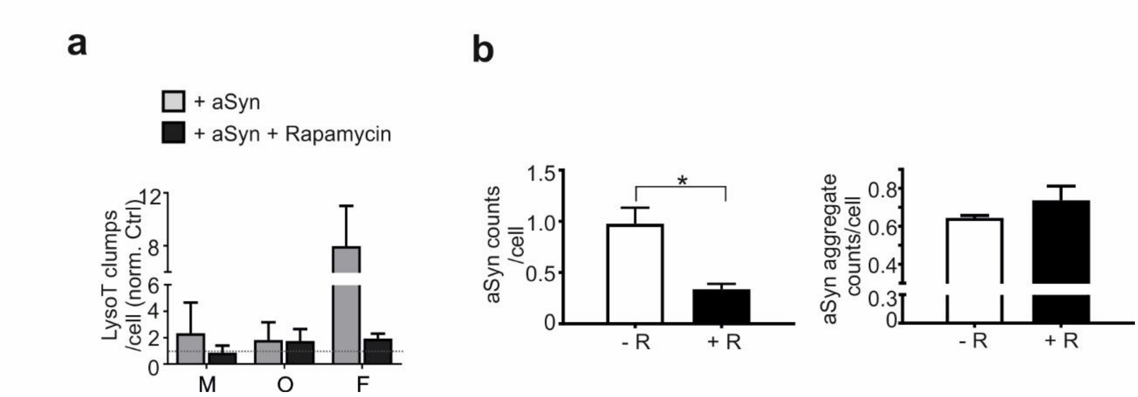

H4 cells were treated with 20 nm rapamycin prior to aSyn application (M: monomers; O: oligomers; and F: fibrils). **a)** ICC analysis of the morphology of lysosomes probed by LysoT. In aSyn-exposed cells, rapamycin pretreatment decreases the amount of larger lysosome clumps/cell induced by extracellular aSyn. Quantification was conducted by normalizing the levels in exposed cells against untreated cells (Ctrl) (n=3, One-way ANOVA). **b)** ICC analysis of extracellular aSyn accumulated in fibril-exposed H4 cells with or without rapamycin (+R or –R) pretreatment. Rapamycin significantly decreases aSyn accumulation as determined by assessing the counts of detectable aSyn signals/cell (left, n=3, One-way ANOVA). However, rapamycin does not reduce the formation of large aSyn aggregates, as determined by assessing the counts of larger aSyn aggregates (area > 2 μm^2)/cell (right, n=3, One-way ANOVA).
